# Uncertainty-Aware Selective Prediction in Brain-Tumour MRI Classification: Selective Risk Reduction and Component-Wise Reliability Findings

**DOI:** 10.3390/bioengineering13101184

**Published:** 2026-10-12

**Authors:** Hala Alshamlan

**Affiliations:** Department of Information Technology, College of Computer and Information Sciences, King Saud University, P.O. Box 51178, Riyadh 11543, Saudi Arabia; halshamlan@ksu.edu.sa

**Keywords:** selective prediction, uncertainty quantification, risk–coverage analysis, error detection, selective classification, brain-tumour MRI, glioma, 3D segmentation, nnU-Net, vision–language models, radiology report generation, BraTS2020

## Abstract

Background: Automated brain-MRI analysis is usually judged by accuracy at a single operating point, yet a model that is accurate on average may still fail without signalling that it is about to fail. Selective prediction addresses this by allowing a model to abstain, but the resulting trade-off between coverage and risk is rarely quantified in medical imaging. Objective: This study asks whether confidence- and entropy-based selective prediction produces a measurable reduction in selective risk in a biomedical vision–language classifier by concentrating errors among withheld cases, and at what coverage cost. Three further components of a brain-MRI-analysis framework—volumetric segmentation, glioma grading and structured report generation—are evaluated as complementary evidence about the reliability profiles of different modules, not as independent contributions. Methods: A LoRA-fine-tuned LLaVA-Med classifier was evaluated on the 1000-image BRISC2025 test partition, with a disjunctive rejection rule based on softmax confidence and predictive entropy. Volumetric segmentation used nnU-Net v2 (3D full-resolution) on BraTS2020 with a three-fold ensemble evaluated on 74 held-out cases; glioma grading (high- versus low-grade) used DenseNet121 on multi-slice representations; report generation used Meta Llama 3.2 3B Instruct fine-tuned with QLoRA on structured findings. All proportions are reported with 95% Wilson score intervals under a single analysis plan documented before the reported analyses were finalised. Results: Withholding 1.80% of predictions (95% CI 1.14–2.83) raised selective accuracy to 97.76% (95% CI 96.63–98.52) and reduced selective risk from 3.40% to 2.24%, a relative reduction of 34.1%. Withheld predictions were strongly enriched for errors (66.7% versus 2.24%; odds ratio 87.3, Fisher exact *p* = 4.6 × 10^−15^), capturing 35.3% of all errors while issuing 99.38% of the predictions that were correct. Considered as a detector of its own errors, the rule has a balanced accuracy of 67.34%; this describes the binary rejection decision at the realised threshold and does not identify the AUROC of the underlying continuous score, which the single operating point leaves anywhere in [0.351, 0.996]. Because the per-sample probability vectors were not retained, the full risk–coverage curve, the rule ablations and calibration error could not be computed, and the thresholds (τ_c_ = 0.6, τ_H_ = 1.0) were selected empirically on an undocumented partition; the selective-prediction finding is therefore reported as a single-operating-point case study and is exploratory. Among the complementary components, three-fold ensembling improved held-out mean Dice from 0.7948 to 0.8150 (+0.0202), reported descriptively because the per-case values needed for a paired test were not preserved. Glioma grading did not achieve better-than-chance discrimination: balanced accuracy 49.91%, Cohen’s κ −0.0023, Matthews correlation coefficient −0.0026, low-grade recall 8.96% (95% CI 5.81–13.57), and accuracy (74.03%) below a constant majority-class classifier (79.46%); the associated McNemar statistic is reported descriptively because slice-level observations are not independent. Report generation produced high automatic similarity scores (BERTScore F1 0.9513) that are not interpretable without baselines. Conclusions: Confidence- and entropy-based abstention produced a measurable reduction in selective risk at low rejection rates, and it did so because errors concentrated among the cases were withheld by the confidence/entropy rule rather than because predictions were discarded indiscriminately. At the realised coverage the rule captured 65.5% (95% bootstrap CI 41.9–87.8) of the reduction achievable by an oracle that rejects only true errors. The component-wise comparison shows that this reliability behaviour cannot be assumed for every module: high aggregate accuracy and useful discrimination came apart in the grading task. These findings are component-wise and do not constitute an end-to-end, externally validated or clinically validated pipeline. The vision–language evaluation is at image level: BRISC2025 carries no patient identifiers, so patient-level independence cannot be established and the reported intervals should be read with that constraint in mind.

## 1. Introduction

Brain-MRI interpretation is central to the diagnosis, treatment planning and follow-up of intracranial tumours, and it is difficult work. Tumour types with distinct biology can look alike on conventional sequences, infiltrative lesions blend into surrounding parenchyma, and the volume of imaging that must be read continues to grow. Interpretation therefore varies between readers, and this variability contributes measurably to diagnostic error [1]. Multi-institutional annotated collections such as BraTS [2] were assembled in part to give automated methods a common footing on exactly these tasks.

Deep learning has changed what automated brain-MRI analysis can do. Self-configuring segmentation frameworks now delineate tumour sub-regions volumetrically with little manual tuning [3], ensembling improves the stability of those delineations [4], and transfer-learned convolutional classifiers separate tumour types with accuracies that were unattainable a decade ago [5]. Large language and vision–language models have extended this reach to the textual side of radiology, raising the prospect of systems that both analyse an image and communicate what they found [6,7].

What has not kept pace is how these systems are evaluated. A model is normally characterised by its accuracy on a held-out partition at a single operating point. That number says how often the model is right; it says nothing about whether the model can tell when it is about to be wrong. The two properties are not the same, and modern networks are systematically overconfident, so a high softmax score is not by itself evidence that a prediction is safe [8]. In a clinical workflow the distinction matters: a system that is right 96% of the time and silent about the remaining 4% imposes a different burden on the reader than one that is right 96% of the time and flags most of its own failures.

Selective prediction—classification with the option to abstain—is the most direct formalisation of that second capability. It dates to Chow’s analysis of the error–reject trade-off [9] and was developed for modern machine learning by El-Yaniv and Wiener [10] and by Geifman and El-Yaniv [11], who established the risk–coverage curve as the appropriate description of a selective classifier. The behaviour is a trade: as the rejection threshold tightens, fewer predictions are issued (coverage falls) and the error rate among those issued (selective risk) should fall with it. A rejection mechanism is worth deploying only if the risk it removes is large relative to the coverage it consumes, and only if the predictions it withholds are genuinely the ones that would have been wrong.

The gap this study addresses is specific. Medical imaging papers that adopt confidence thresholding typically report accuracy before and after rejection at one threshold, without the coverage figure beside it, without an interval, and without any test of whether rejected cases are actually enriched for errors. Reported that way, the improvement is uninformative: accuracy among retained predictions can always be raised by discarding enough of them. What is missing is a joint account of coverage, selective accuracy, selective risk, error-capture rate and rejection enrichment, with uncertainty intervals, so that a reader can judge whether the reduction in selective risk justifies the coverage cost. Work that does treat abstention as a first-class object—for example the Dempster–Shafer fail-safe framework applied to fetal brain-MRI segmentation [12]—remains uncommon, and ensemble-based uncertainty estimation [13] is often too expensive to deploy at the scale a clinical service requires.

This study places that question at its centre and evaluates three further components of the same brain-MRI-analysis framework alongside it, for a specific reason: reliability is not a property that one module can certify on behalf of a whole system. Volumetric segmentation is included because it supplies the spatial characterisation—tumour sub-region extent and location—on which any downstream description depends. Glioma grading is included because it is a second clinically meaningful prediction task on the same imaging collection, and therefore a test of whether the reliability behaviour observed in one classifier generalises to another. Structured report generation is included because it is where analytical output becomes something a clinician reads, and where the consequences of an unflagged error are realised. Together they characterise what different modules of an MRI-analysis framework can and cannot reliably deliver.

**Figure 1:**
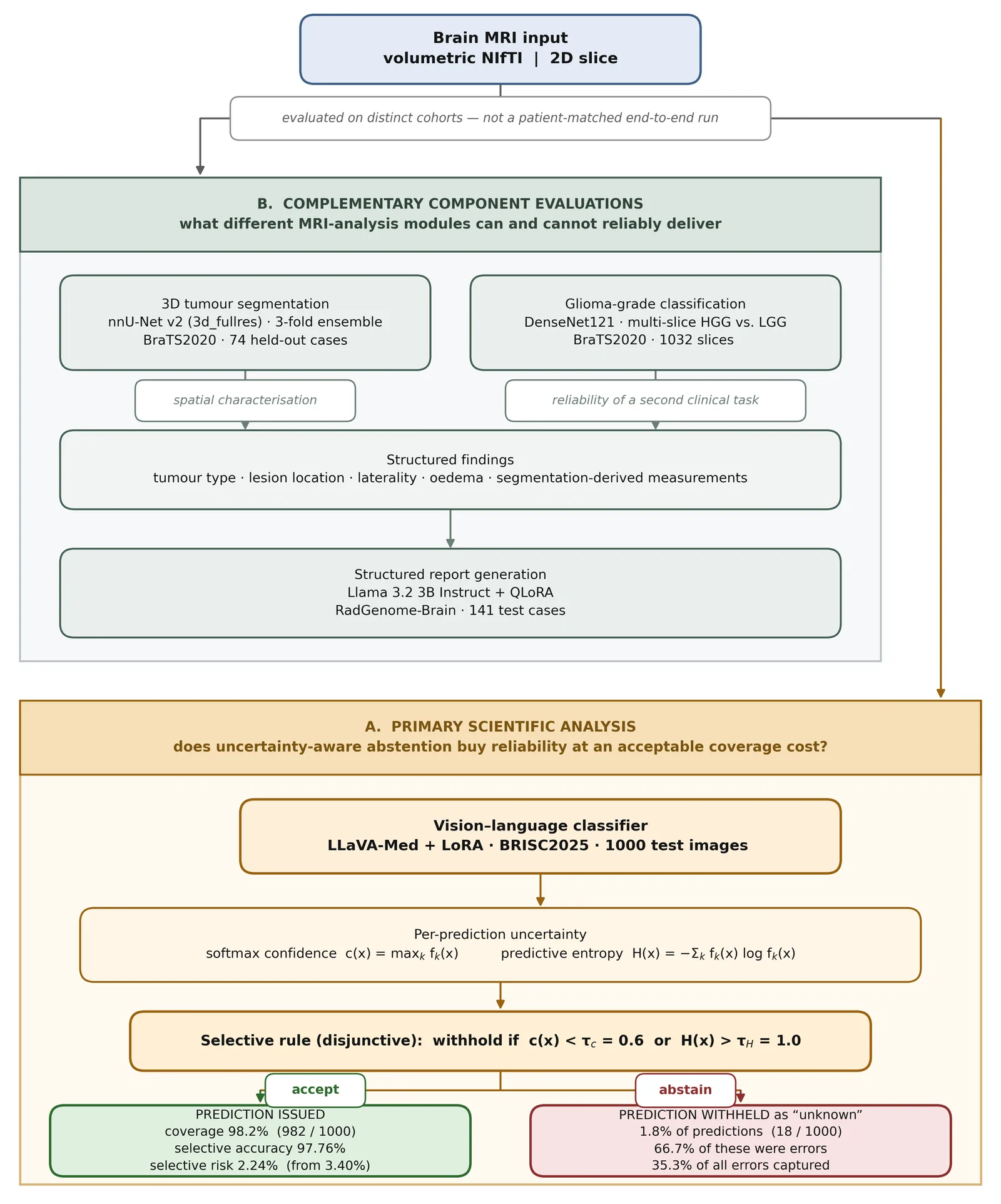
Conceptual architecture of the brain-MRI-analysis framework and the scientific hierarchy of this study. Panel (**A**) (primary scientific analysis) shows the uncertainty-aware selective-prediction branch: the LoRA-fine-tuned LLaVA-Med classifier, the per-prediction confidence and entropy computed from its output distribution, the disjunctive rejection rule, and the two possible outcomes with the results obtained at the operating threshold. Panel (**B**) (complementary component evaluations) shows volumetric segmentation and glioma grading supplying structured findings to the report generator. Arrows denote the conceptual flow of information between stages, not an executed pipeline: the four components were trained and evaluated on the distinct cohorts named beneath each block (see Table 1), and were never evaluated as a single patient-matched end-to-end system. No end-to-end, external or clinical validation is reported in this manuscript.

These are component-wise experiments. Each component was developed and evaluated on its own cohort, using inputs of the form the corresponding stage expects, and no experiment passed a single set of patient cases through segmentation, grading, report generation and selective prediction in sequence. The relationship drawn between the components in Figure 1 is a conceptual architecture, not a validated pipeline. This manuscript therefore reports neither end-to-end validation nor external validation nor clinical validation, and it does not claim them.

Research question. Does confidence- and entropy-based selective prediction improve the reliability of a fine-tuned biomedical vision–language classifier by concentrating erroneous predictions among withheld cases, and is the resulting reduction in selective risk large relative to the coverage it costs?

**Hypothesis.** 
*Confidence- and entropy-based selective prediction improves the reliability of a biomedical vision–language classifier by concentrating erroneous predictions among rejected, uncertain cases, thereby reducing selective risk at a limited coverage cost.*

Primary contribution. A single-operating-point case study of uncertainty-aware selective prediction in which coverage, selective accuracy, selective risk, error-capture rate and rejection enrichment are reported jointly for one threshold pair, with interval estimates, exact oracle and random reference points, and an explicit statement of the validation experiment that the preserved records could not support.

Secondary contributions. (i) A comparison of three volumetric segmentation architectures on BraTS2020 with a three-fold ensemble evaluated on a held-out cohort, reported descriptively where the preserved data do not support inference. (ii) A negative result for volumetric glioma grading, established with chance-corrected statistics and a formal comparison against a constant-output baseline, which shows that aggregate accuracy can conceal the absence of usable discrimination. (iii) A single analysis plan, documented before the reported analyses were finalised and applied uniformly to every component, including the cases in which the available data did not support a test and a previously reported test was withdrawn.

Scope. The claims made here are bounded by the evaluation design. No component was tested on data originating outside the collection that supplied its training data; the glioma-grading partition was constructed at slice rather than patient level; BRISC2025 carries no patient identifiers, so the vision–language evaluation is at image level and patient-level independence cannot be established; random seeds were not fixed, so every reported figure describes one experimental configuration rather than a distribution over configurations; and no practising radiologist evaluated any output under a blinded protocol. These boundaries are maintained in the Abstract, Methods, Results, Discussion, Limitations and Conclusions.

**Table 1:** Dataset, partition, unit of analysis and study role for each experimental component.

Component	Dataset	Training Partition	Evaluation Partition	Unit of Analysis	Role in this Study
Vision–language classification and selective prediction	BRISC2025	5000 images	1000 images	Image	Primary scientific analysis (Section 4.2)
3D tumour segmentation	BraTS2020	~281 cases (3 folds)	74 held-out cases	Patient	Complementary: spatial tumour characterisation (Section 4.4)
Glioma-grade classification	BraTS2020 (multi-slice)	Not preserved (Section 3.7)	1032 slices	Slice (see Section 6)	Complementary: reliability of a second clinical task (Section 4.6)
Structured report generation	RadGenome-Brain	487 cases	141 cases	Case	Complementary: communication of structured findings (Section 4.7)

### Methodological Contribution in Relation to Existing Techniques

The components used here are established methods and are not claimed as novel. nnU-Net [3], LoRA [14], QLoRA [15], LLaVA-Med [7], fold ensembling, softmax confidence, predictive entropy and threshold-based rejection all pre-date this work, and the study makes no architectural modification to any of them. What is contributed is the evaluation framework applied to them and the analysis it yields.

Concretely, the contribution is four-fold. First, the primary outcome of this study is reliability under abstention rather than accuracy at a fixed operating point, and the reporting is structured accordingly: coverage and selective risk are always given together, and the denominator of every reported accuracy is stated. Second, the rejection mechanism is characterised by whether it withholds the right predictions, using error-capture rate and rejection enrichment with an explicit test of association, rather than by the accuracy improvement alone. Third, the same evidentiary standard is applied to every component, which is what makes the heterogeneity of the results interpretable: the positive selective-prediction finding, the descriptive segmentation difference and the negative grading result are all products of one analysis plan, not of four different ones. Fourth, the study reports what its records do not support—a withdrawn statistical test, three irreconcilable accuracy figures for one model, undocumented threshold selection—rather than presenting only the components that worked.

## 2. Related Work

### 2.1. Selective Prediction and Uncertainty Quantification

Selective prediction, or classification with a reject option, originates with Chow’s analysis of the error–reject trade-off [9] and was formalised for modern machine learning by El-Yaniv and Wiener [10] and by Geifman and El-Yaniv [11]. The central object in that formalisation is not accuracy at a single threshold but the function relating coverage to selective risk across all thresholds, from which any operating point can be read.

Two results bear directly on rejection rules built from softmax outputs. Guo et al. [8] showed that modern neural networks are systematically overconfident, which means a confidence-based rule inherits whatever miscalibration the underlying model exhibits; calibration analysis is therefore a necessary companion to any such scheme rather than an optional extra. Lakshminarayanan et al. [13] proposed deep ensembles as a stronger uncertainty estimator, at a cost in training and inference that is often prohibitive in practice, and whether single-model confidence and entropy approach ensemble-quality uncertainty in medical imaging remains open.

Applications of abstention in medical imaging are comparatively few. Fidon et al. [12] applied a Dempster–Shafer trustworthy framework to fetal brain-MRI segmentation across 540 scans from 13 centres and showed that an explicit fail-safe mechanism improved rather than merely constrained nnU-Net Dice (90.4% to 91.1%), which established that a trust layer need not cost performance. That work concerns segmentation; the equivalent question for a vision–language classifier—whether abstention removes a disproportionate share of the errors, and at what coverage cost—is the question addressed here.

### 2.2. Volumetric Segmentation and Glioma Grading

Isensee et al. [3] introduced nnU-Net, a self-configuring segmentation framework that adapts preprocessing, patch size and network topology to a given dataset, achieving leading performance across most tasks of the Medical Segmentation Decathlon. Henry et al. [4] proposed a self-ensembled, deeply supervised 3D U-Net for the BraTS 2020 challenge, reporting Dice scores of 0.81, 0.91 and 0.85 for enhancing tumour, whole tumour and tumour core respectively, and demonstrating the value of ensembling for segmentation robustness.

For glioma grading, Rasheed et al. [5] applied DenseNet121-based transfer learning to multi-class brain-tumour classification, and Yang et al. [16] used transfer learning on conventional MR images to distinguish high- from low-grade glioma. Wen et al. [17] proposed a joint segmentation-and-grading framework and identified limited grading-label availability, rather than segmentation itself, as the harder sub-problem—a pattern consistent with the pronounced HGG/LGG imbalance in the BraTS2020 cohort used here. Class imbalance is known to bias medical imaging classifiers systematically towards the majority class [18], which is one reason aggregate accuracy is a poor summary of grading performance and why the analysis in Section 4.7 is built on chance-corrected statistics instead.

### 2.3. Grounded Report Generation

Lei et al. [19] introduced AutoRG-Brain, a grounded report-generation system for brain MRI that couples anomaly segmentation with transformer-based generation, released alongside the RadGenome-Brain-MRI dataset, and showed that grounding generation in validated visual evidence improved report quality relative to junior-radiologist baselines. Chu et al. [20] showed that visual retrieval-augmented generation reduces, without eliminating, hallucination in medical multimodal large language models, which indicates that grounding strategies constrain the problem rather than solve it. Parameter-efficient fine-tuning through LoRA [14] and its quantised extension QLoRA [15] made adaptation of such models feasible on modest hardware, and Voinea et al. [21] used this route to fine-tune a Llama 3 8B model for radiology conclusion generation on a large corpus of real hospital reports, providing the closest published point of comparison for the report-generation component reported in Section 4.8.

### 2.4. Position of the Present Study

Taken together, the literature supplies strong architectures for each task and a well-developed theory of selective classification, but little medical imaging work that reports the full accounting a rejection mechanism requires, and less that asks whether the reliability behaviour of one module can be assumed for the others in the same system. This study supplies the first of those for a fine-tuned biomedical vision–language classifier and, through the complementary components, provides evidence bearing on the second.

## 3. Materials and Methods

### 3.1. Study Design and Scientific Objective

The study has one primary scientific objective and three subordinate ones. The primary objective is to determine whether uncertainty-aware selective prediction improves the reliability of a fine-tuned biomedical vision–language classifier, and to quantify the reduction in selective risk against the coverage it costs. This is the analysis reported in Section 4.2, and it is the analysis the hypothesis in Section 1 concerns.

The subordinate objectives characterise three further components of the same MRI-analysis framework: the accuracy of volumetric tumour segmentation and the effect of fold ensembling on it (Section 4.5); the discriminative behaviour of a volumetric glioma-grade classifier under chance-corrected evaluation (Section 4.7); and the automatic text-similarity behaviour of a structured-finding-grounded report generator (Section 4.8). These are not presented as four equal contributions. They are complementary evaluations that establish the standalone behaviour of each module and, taken with the primary analysis, support the broader observation that reliability profiles differ substantially between modules of a single framework.

The design is component-wise by construction. Each component was trained and evaluated on the cohort appropriate to its task and its input format, and the components were never run in sequence over a shared set of patients. This design permits attribution of performance to individual stages, which an end-to-end metric would obscure—a point borne out by the grading result, where a single pipeline figure would have hidden which stage was failing. It does not permit any statement about end-to-end behaviour, and none is made. The end-to-end experiment that would be required is specified in Section 7.

### 3.2. Datasets and Evaluation Cohorts

Three public collections were used. Table 1 states, for each component, the dataset, the training and evaluation partitions, the unit of analysis and the role the component plays in the study.

BraTS2020 [2] comprises 369 multimodal MRI scans with co-registered T1, T1CE, T2 and FLAIR modalities, expert-annotated masks for necrotic core/non-enhancing tumour (NCR/NET), oedema (ED) and enhancing tumour (ET), and case-level HGG/LGG labels. Approximately 355 cases with usable annotations were used for segmentation. The scans were acquired across multiple contributing institutions; the training and held-out partitions used here are random partitions of this pooled collection rather than a partition that holds out one institution, which is why the held-out cohort does not test cross-institutional domain shift (Section 6).

BRISC2025 comprises 6000 T1-weighted 2D MRI slices across four classes (glioma, meningioma, pituitary tumour, no tumour), partitioned by the dataset providers into 5000 training and 1000 test images. The vision–language classifier and its selective-prediction layer were evaluated on the 1000-image test partition (glioma 254, meningioma 306, no tumour 140, pituitary 300).

RadGenome-Brain MRI [19] was used for report-generation fine-tuning. After removing subsets unrelated to tumour radiology reporting (ISLES22, WMH) and retaining the BraTS-GLI, BraTS-MEN and BraTS-MET subsets, the filtered dataset comprised 697 cases split into 487 training, 69 validation and 141 test samples, using the dataset’s predefined patient-level split.

The BraTS-GLI subset of RadGenome-Brain and the BraTS2020 collection both derive from the BraTS glioma cohorts. Patient-level overlap between the report-generation fine-tuning set and the segmentation cohort therefore cannot be excluded from the information available to us, and is recorded as a limitation in Section 6. Because the two components are evaluated independently and no result is transferred between them, this does not affect the validity of either result taken alone.

### 3.3. Overall Framework and Component Relationships

Figure 1 sets out the relationship between the components and separates, visually and explicitly, the primary scientific analysis from the complementary component evaluations. Panel A contains the uncertainty-aware selective-prediction branch: a vision–language classifier produces a class distribution, from which softmax confidence and predictive entropy are computed, and a disjunctive selective rule either issues the prediction or withholds it as “unknown”. Panel B contains the complementary components: volumetric segmentation and glioma grading operate on the imaging volume and feed structured findings—tumour type, lesion location and characteristics, laterality, oedema presence and segmentation-derived measurements—into the report generator, which converts them into a radiology-style impression.

Two readings of this figure must be kept apart. The arrows describe a conceptual architecture: they state which output would supply which input if the framework were assembled and run as one system. They do not describe an experiment. Each block was trained and evaluated on its own cohort, listed beneath it in the figure and in Table 1, and no set of patient cases traversed the framework from input to impression. In particular, the structured findings used to fine-tune and evaluate the report generator were the annotation-derived findings supplied with RadGenome-Brain, not findings produced by the segmentation and grading models evaluated here. The performance of any block therefore carries no information about the performance of the assembled framework, since errors made upstream were never propagated downstream in any experiment reported in this manuscript.

### 3.4. Vision–Language Classification

The classifier is a LoRA-fine-tuned LLaVA-Med model [7,14] adapted to the four-class BRISC2025 labelling task. Fine-tuning used a previously validated LoRA adapter rather than training from scratch, with glioma-class oversampling and brightness, contrast and small-rotation augmentation, a learning rate of 5 × 10^−6^, gradient accumulation of 8, and one epoch. The low learning rate and single epoch were chosen to preserve the stability of the pre-trained biomedical representation.

### 3.5. Uncertainty-Aware Selective Prediction

Let f(x) denote the model’s softmax output over K = 4 classes for input x. Confidence is defined as c(x) = max_k_ f_k_(x) and predictive entropy as H(x) = −Σ_k_ f_k_(x) log f_k_(x), the standard Shannon entropy of the output class-probability vector. A selective classifier is the pair (f, g), where g is a binary selection function: a prediction is issued when g(x) = 1 and withheld and labelled “unknown” otherwise [10,11]. The rule evaluated here is a logical disjunction: g(x) = 0 if c(x) < τ_c_ or H(x) > τ_H_, and 1 otherwise, with τ_c_ = 0.6 and τ_H_ = 1.0. The disjunction was chosen so that either form of uncertainty—a low winning probability, or probability mass spread across several classes—is sufficient to trigger abstention.

Coverage is the proportion of inputs for which a prediction is issued, and selective risk is the error rate among issued predictions. These two quantities jointly characterise the method, and reporting either alone is uninformative, since coverage can always be traded for risk. Two further quantities describe whether the mechanism withholds the right predictions: the error-capture rate, the proportion of all errors that fall among withheld predictions, and rejection enrichment, the ratio between the error rate among withheld predictions and the error rate among issued ones.

The threshold pair τ_c_ = 0.6 and τ_H_ = 1.0 requires careful statement, because the principal finding of this study is based on this. These values were chosen empirically during development. They were not optimised against a validation objective, no search over the threshold space was carried out, and they are not claimed to be optimal in any sense; they are one operating point at which the rule was evaluated. The partition on which they were chosen is not documented in the surviving experimental records, and the possibility that they were chosen with reference to the test partition cannot be excluded. Were that the case, the operating point reported in Section 4.2 would be optimistically biased, and the magnitude of that bias cannot be estimated from the available data.

A second limitation follows from the first. No sensitivity analysis over alternative thresholds was carried out, so the reported result characterises one point on the risk–coverage curve rather than the curve itself, and the reduction in selective risk cannot be shown to be robust to the choice of threshold. The protocol that would resolve both limitations—selecting thresholds on an independent validation split with the test partition left untouched, then reporting the full risk–coverage curve together with the rule ablations and calibration metrics—is specified in Section 7 and requires a single inference pass and no retraining. Until it has been carried out, the finding reported in Section 4.2 should be read as supportive but provisional.

Robustness was evaluated under four controlled perturbations (brightness decrease, brightness increase, rotation left, rotation right) applied to ten base images, with a prediction considered stable if its label was unchanged relative to the unperturbed image.

### 3.6. 3D Tumour Segmentation

Volumetric segmentation used nnU-Net v2 [3] in its 3d_fullres configuration, which determines preprocessing, patch size and normalisation automatically from the dataset fingerprint. BraTS2020 volumes and masks were reorganised into the nnU-Net directory structure with each of the four modalities assigned a separate channel identifier; the original BraTS label 4 was remapped to label 3 for compatibility with the sequential labelling convention. Three independent cross-validation folds were trained, with checkpoint selection based on the exponential moving average (EMA) pseudo-Dice achieved during internal validation. The ensemble combines the three folds by averaging predicted voxel-wise softmax class probabilities and taking the arg-max per voxel, the standard rule implemented by nnUNetv2_predict when multiple fold checkpoints are supplied [3]; no custom weighting was applied. SegResNet and a standard 3D U-Net were trained under comparable settings as baselines. Training followed the nnU-Net v2 default automated configuration without manual hyperparameter tuning.

Three of five folds were trained, for compute reasons. This is not five-fold cross-validation and is not described as such; the ensemble is a three-fold ensemble, and the fold-to-fold variability that can be estimated from it is correspondingly limited.

### 3.7. Glioma-Grade Classification

Volumetric glioma-grade classification distinguishing HGG from LGG used multi-slice representations extracted from BraTS2020 volumes. DenseNet121, ResNet50 and a single-channel 3D CNN were evaluated under comparable preprocessing and training settings, with early stopping and regularisation. The pipeline operates on multi-slice 2D representations sampled from each volume rather than a native 3D convolutional input; “volumetric” describes the case-level prediction target rather than the network architecture.

A reproducibility limitation for this component is stated directly. The number of slices sampled per volume, the slice-selection criterion, and the rule used to aggregate slice-level outputs into a case-level prediction were not preserved in the experimental logs, and cannot be reported. This component is therefore not independently reproducible from the description given here, and its results are not presented as a reproducible benchmark. It is reported in Section 4.7 as a negative result—a demonstration that a standard architecture fails on this task under honest evaluation—and the protocol required to re-establish it properly is specified in Section 7.

The partition used for this component was constructed at the slice level rather than the patient level, so slices from a single patient may appear on both sides of the partition. The consequences are analysed in Section 4.7 and Section 5.4. A patient-level re-run is specified in Section 7.

### 3.8. Structured Report Generation

Report generation used Meta Llama 3.2 3B Instruct fine-tuned via QLoRA [15] with 4-bit NormalFloat (NF4) quantisation on a Tesla T4 GPU. Rather than interpreting MRI pixels directly, the pipeline followed a fixed sequence: MRI scan → classification and segmentation models → structured findings (suspected tumour type, lesion location and characteristics, oedema presence, laterality, segmentation-derived measurements) → LLM → radiology impression. Task scope was restricted to impression generation, excluding treatment recommendation and independent diagnostic decision-making. Fine-tuning ran for 2 epochs with a maximum sequence length of 1024 tokens. The remaining fine-tuning parameters are given in Table 2.

The learning rate used for this QLoRA run was not preserved in the experimental logs and cannot be reported; this is recorded as a reproducibility limitation in Section 6.

Generated outputs were reviewed during development by the research team to identify unsupported content. This was an informal developmental check, not a blinded evaluation by practising radiologists, and it is reported as a scoping step rather than as validation of clinical adequacy.

### 3.9. Evaluation Metrics

Segmentation was quantified using the Dice similarity coefficient. The aggregation convention is stated explicitly, because it differs from the BraTS challenge convention and this affects comparability with published BraTS results. The mean Dice reported in Section 4.5 is the arithmetic mean of the Dice coefficients for the three raw label channels—NCR/NET, ED and ET—and is not the mean over the nested composite regions (enhancing tumour ET, tumour core TC = NCR/NET ∪ ET, and whole tumour WT = NCR/NET ∪ ED ∪ ET) used by the BraTS challenge and by the studies cited in Section 2.2. Values computed under the two conventions are not directly comparable, and no numerical comparison with published BraTS challenge results is made anywhere in this manuscript. Recomputation under the ET/TC/WT convention, which requires no retraining, is specified in Section 7.

Classification was quantified using accuracy, per-class precision, recall and specificity, macro-F1, balanced accuracy, Cohen’s κ and the Matthews correlation coefficient (MCC). For imbalanced tasks, accuracy is never reported as a primary outcome and is always accompanied by a majority-class baseline with its confidence interval, following the argument of Chicco and colleagues [22,23] that MCC is more reliable than accuracy or F1 in skewed confusion-matrix settings. Selective classification was quantified using coverage, selective accuracy, selective risk, error-capture rate and rejection enrichment. Report generation was quantified using BERTScore [24] and ROUGE [25] against reference impressions.

### 3.10. Statistical Analysis

The analysis plan set out below was documented before the analyses reported in Section 4 were finalised, and was applied uniformly to every component. It was not registered prospectively and was not fixed before model development or before the selective-prediction thresholds were chosen. It is an internal analysis protocol rather than a preregistration, and the term “pre-specified” is deliberately avoided for that reason.

Unit of analysis. The patient is the intended unit of analysis. Where an evaluation could not be aggregated to patient level—specifically the glioma-grading component (Section 4.7) and the image-level vision–language evaluation (Section 4.2 and Section 4.4)—results are reported descriptively and the non-independence is stated at the point of reporting.

Interval estimation. Proportions are reported with 95% Wilson score intervals [26], which are preferred to Wald intervals because several reported proportions exceed 0.97, where the Wald interval is unreliable. Continuous metrics are reported with bias-corrected and accelerated (BCa) bootstrap intervals using 10,000 resamples. Patient-level resampling is the intended design, but it could not be applied to the two components for which patient identifiers were unavailable: the vision–language evaluation is resampled at image level and the grading statistics at slice level. Intervals from those two components are therefore conditional calculations under an unverified independence assumption, and are labelled as such at the point of reporting in Section 4.3 and Section 4.7. Composite metrics (macro-F1, κ, MCC, balanced accuracy) are bootstrapped by recomputing the metric within each resample.

Hypothesis testing. Paired categorical comparisons on the same samples use McNemar’s test. Independent proportions use Fisher’s exact test. Paired continuous comparisons use the Wilcoxon signed-rank test with a paired BCa bootstrap of the mean difference. Multi-arm comparisons use the Friedman test followed by pairwise Wilcoxon tests with Holm–Bonferroni correction. The significance threshold is α = 0.05, two-sided, applied within each pre-defined family of comparisons.

Effect sizes. Every *p*-value is accompanied by an effect size: Cohen’s d_z_ and the raw mean difference for paired continuous comparisons, Cliff’s delta for non-parametric comparisons, and absolute and relative risk differences with intervals for proportions.

Class imbalance and rejected predictions. Every result on an imbalanced task is accompanied by a majority-class baseline and its interval. Rejected (“unknown”) predictions are handled by reporting coverage and selective risk jointly; they are never silently removed from a denominator, and the denominator of every reported accuracy is stated explicitly.

Tests not performed. No test is computed from values reconstructed from a published figure, or from aggregate means in the absence of per-case data. Where patient-level independence could not be verified, statistics are still reported—the image-level selective-prediction analysis in Section 4.3 and the slice-level grading comparison in Section 4.7—but they are presented as conditional calculations under an unverified independence assumption and are labelled descriptive rather than confirmatory at the point of reporting. No inferential claim about a patient population is drawn from them. Where a comparison is descriptive, it is labelled descriptive. Section 4.6 documents a case in which a previously reported test was withdrawn on these grounds.

### 3.11. Reproducibility, Stochastic Variability and Implementation

All training was performed on Google Colab using a Tesla T4 GPU. Segmentation used nnU-Net v2 in the 3d_fullres configuration with the framework’s automated settings throughout: framework-determined patch and batch size, SGD with the default poly learning-rate schedule, the default augmentation pipeline, and checkpoint selection by the highest EMA pseudo-Dice on internal validation. Three of five folds were trained. Report-generation fine-tuning used PEFT with BitsAndBytes NF4 quantisation and the configuration in Table 2. The vision–language classifier was fine-tuned with LoRA as described in Section 3.4. Glioma-grade classification used DenseNet121, ResNet50 and a single-channel 3D CNN under matched preprocessing with early stopping and regularisation.

Deep learning results are not deterministic. Weight initialisation, data partitioning, mini-batch sampling order, stochastic augmentation, non-deterministic GPU kernel scheduling and library version differences all contribute variance to the final metrics, and the magnitude of that variance is a property of the training configuration that has to be measured rather than assumed. Reporting a single run therefore gives a point estimate whose sampling distribution is unknown, and two configurations that differ by less than that unknown spread cannot be distinguished from one another. This has been demonstrated repeatedly outside medical imaging: in automated fault detection and diagnosis for building air-handling units, self-supervised and semi-supervised time-series models are evaluated across repeated randomised runs precisely because single-run performance is not a stable characterisation of a method [27,28]. The mechanism is not domain-specific, and there is no reason to expect brain-MRI models to be exempt from it.

Random seeds were not fixed or recorded for any component in this study, and library versions were not systematically logged. Each component was therefore trained under a single uncontrolled initialisation, and exact numerical reproduction of the reported figures should not be expected even from identical code and data. This is stated plainly rather than as a general caveat, because it bounds what the reported point estimates can be taken to mean: each describes one experimental configuration and not a distribution over configurations. Repeated-seed evaluation with mean ± standard deviation reporting was not performed and is not claimed; it is specified in Section 7 as a required next step. The consequences for the interpretation of each component are set out in Section 5.6, and the limitation is recorded in Section 6.

To make the boundary between what is and is not reproducible explicit, the record is itemised here. Preserved and reported: the vision–language fine-tuning configuration (LoRA, learning rate 5 × 10^−6^, gradient accumulation 8, one epoch, oversampling and augmentation scheme); the nnU-Net v2 3d_fullres configuration and the number of folds trained; the report-generation configuration in Table 2 except its learning rate; the dataset partitions as supplied by the dataset providers for BRISC2025 and RadGenome-Brain; all aggregate metrics and the confusion matrices from which the class-aware statistics reported in the Results are computed. Not preserved and therefore not reportable: random seeds and library versions for every component; the partition on which the selective-prediction thresholds were chosen; per-sample probability vectors and per-image predictions for the vision–language component; per-case Dice values for the segmentation cohort; the slice count, slice-selection criterion and case-level aggregation rule for glioma grading; the patient-level partition for glioma grading, which was constructed at slice level; and the learning rate of the report-generation run. The public repository described below will contain the items in the first group; it cannot contain the items in the second, and no undertaking is given that it will.

All code, configuration files, dataset partition files, derived metric tables and statistical analysis scripts will be deposited in a public repository with a permanent identifier prior to publication. Availability on request is not considered sufficient for a study of this kind.

## 4. Results

### 4.1. Validation Design and Unit of Analysis

Table 1 states the dataset, partition, unit of analysis and study role for each component. Three features of this design constrain the interpretation of everything that follows and are stated here rather than in the Limitations alone. First, no component was evaluated on data originating outside the collection from which its training data was drawn; the 74-case segmentation cohort is an official held-out split of the same pooled BraTS2020 collection, not a separate site or scanner population, so no result in this manuscript constitutes external validation. Second, the glioma-grading partition was constructed at slice rather than patient level, so its figures are not generalisation estimates. Third, the vision–language evaluation is at the level of individual images rather than patients; BRISC2025 does not carry patient identifiers that would permit patient-level aggregation, and the reported intervals therefore treat images as independent units.

### 4.2. Primary Analysis: Uncertainty-Aware Selective Prediction

Prior to rejection, the LoRA-fine-tuned LLaVA-Med classifier achieved 96.60% accuracy on the 1000-image test partition (966/1000; 95% CI 95.29–97.56), with a macro-F1 of 96.96% and Cohen’s κ of 0.954. Per-class results with intervals are given in Table 3. This is the operating characteristic the selective layer is applied to, and it is reported first because selective risk is only interpretable against the baseline risk it is compared with.

**Figure 2:**
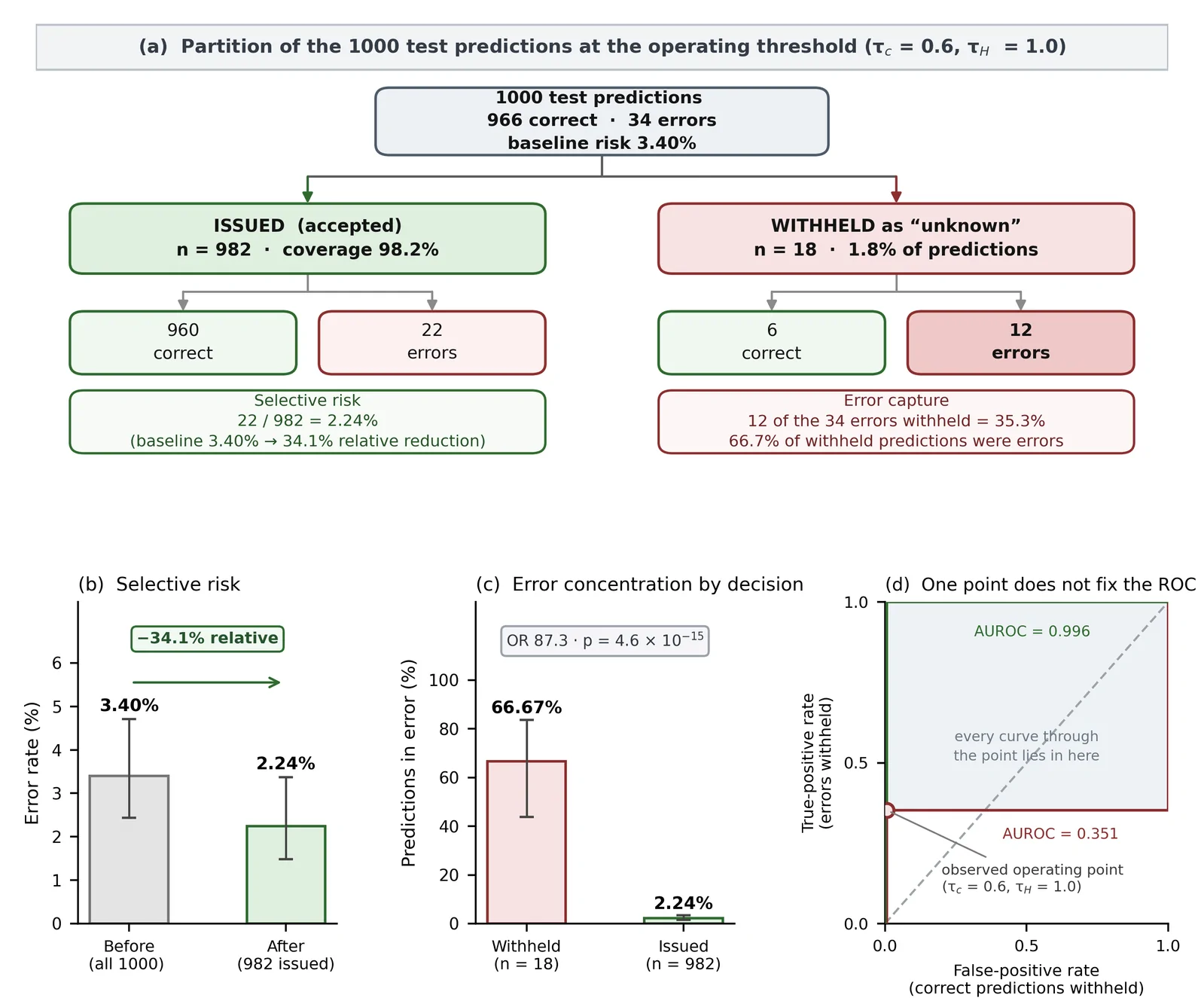
Uncertainty-aware selective prediction on the 1000-image BRISC2025 test partition. (**a**) Partition of the test predictions by the disjunctive rejection rule: of the 34 errors present before rejection, 12 fall among the 18 withheld predictions and 22 remain among the 982 issued ones. (**b**) Error rate before and after rejection, with 95% Wilson score intervals; the denominators differ (1000 before, 982 after) and are stated in the axis labels. (**c**) Proportion of predictions in error among withheld versus issued predictions, with 95% Wilson score intervals; the difference is the rejection-enrichment result. (**d**) Why the single operating point does not determine the discriminative value of the underlying uncertainty score. The two step curves are the extremal receiver-operating-characteristic curves consistent with the observed point: both pass through it, but one has area 0.351 and the other 0.996. Every curve through that point lies in the shaded region. The observed point therefore does not identify the continuous-score AUROC and does not justify the lower bound of 0.673 claimed in the previous version; the only bounds it supports are the extremal values shown, 0.351 and 0.996. Panels (**a**,**c**) together show that the observed reduction in selective risk arises because errors concentrate among withheld cases, not because predictions were discarded at random. All values describe a single operating point, selected empirically on an undocumented partition; the full risk–coverage curve could not be computed from the preserved records (Section 4.3).

Applying the disjunctive rejection rule of Section 3.5 withheld 1.80% of predictions (18 of 1000; 95% CI 1.14–2.83), leaving a coverage of 98.2%. Table 4 reports the resulting selective-prediction behaviour and Figure 2 shows how the 1000 predictions are partitioned by the rule. Selective accuracy—accuracy among issued predictions—was 97.76% (960/982; 95% CI 96.63–98.52), and selective risk fell from 3.40% (95% CI 2.44–4.71) to 2.24% (95% CI 1.48–3.37), an absolute reduction of 1.16 percentage points and a relative reduction of 34.1%.

The result that bears on the hypothesis is not the accuracy improvement but where the errors went. Of the 34 errors present before rejection, 12 fell among the 18 withheld predictions, so the mechanism captured 35.3% of all errors (95% CI 21.49–52.09) while declining fewer than one prediction in fifty. Withheld predictions were substantially enriched for errors: 66.7% of them were errors (95% CI 43.75–83.72), against 2.24% of issued predictions, an enrichment factor of approximately 30 (Fisher exact test, odds ratio 87.3, *p* = 4.6 × 10^−15^). Errors were therefore not distributed uniformly across the confidence range; they concentrated in the region the rule was designed to isolate, which is the behaviour required for abstention to be useful rather than merely arithmetically favourable.

The derivation of Table 4 is stated explicitly, because the denominator convention determines the values and because the previous version of this manuscript reported these quantities in a form that obscured it. The evaluation recorded three aggregate figures: accuracy before rejection (96.6%), accuracy after rejection (96.0%), and rejection rate (1.8%). These are mutually consistent only under the convention that the post-rejection figure uses the full 1000-image denominator with withheld predictions counted as non-correct; reading it instead as accuracy among the 982 issued predictions would require 23.3 correct predictions within the 18 that were withheld, which is not possible. Adopting the consistent convention, 12 of the 18 withheld predictions were errors and 6 were correct, from which every quantity in Table 4 and Figure 2 follows arithmetically. The convention is reported so that any reader can verify the derivation from the three recorded figures.

Mean prediction confidence was 0.9639 with mean entropy 0.1111; the minimum observed confidence was 0.3859 and the maximum entropy 1.2560. The withheld predictions therefore occupied a distinguishable region of the confidence–entropy space rather than being distributed throughout it, which is consistent with the enrichment result but is a descriptive observation rather than a separate test.

Table 4 characterises a single operating point rather than the full risk–coverage trade-off. This is a limitation of the present analysis rather than a property of the method: the complete curve, together with a rejection-rule ablation and a calibration analysis of the kind Guo et al. [8] showed to be necessary for confidence-based rules, is specified in Section 7 as the principal outstanding analysis. It requires a single inference pass storing per-sample softmax vectors and no retraining.

### 4.3. Error-Detection Characteristics of the Rejection Rule

Reviewers of the previous version asked for the full risk–coverage curve, ablations of the confidence and entropy criteria, calibration metrics, and error-detection AUROC and AUPRC. Those analyses require the per-sample softmax vectors, which were not preserved (Section 3.11), and they are therefore not reported. What the preserved records do determine exactly is the 2 × 2 partition in Figure 2a, and that partition is sufficient to characterise the rejection rule as a binary detector of its own errors. This section reports what follows from it and states precisely where the derivation stops.

Treating “withheld” as a positive detection of an impending error, the rule achieves a sensitivity of 35.29% (95% CI 21.49–52.09) and a specificity of 99.38% (95% CI 98.65–99.72). The asymmetry is the operationally relevant feature: the rule withholds about one error in three, but it does so while issuing 99.38% of the predictions that were in fact correct, so the reduction in selective risk is not obtained by indiscriminate abstention. Youden’s J is 0.347 and the balanced accuracy of the detector is 67.34%. Precision among withheld predictions is 66.67% against a base error rate of 3.40%, a lift of 19.6-fold. Table 5 collects these quantities.

Two further quantities are reported descriptively rather than as estimates of a population parameter. First, considered as a detector of its own errors, the rejection rule withheld 35.29% of the errors, which is its sensitivity, while issuing 99.38% of the correct predictions, which is its specificity. The balanced accuracy of that binary decision is the mean of sensitivity and specificity, (0.3529 + 0.9938)/2 = 0.6734; that is 67.34%. This is a property of the binary rejection rule at the single realised threshold pair. It is not a bound on, and not an estimate of, the area under the receiver-operating-characteristic curve of the underlying continuous uncertainty score. A previous version of this manuscript treated the area under the piecewise-linear path through the observed point as such a bound; that inference is invalid, because the ROC curve of a continuous score need not be concave and so need not lie above that path. Rankings consistent with the observed operating point admit continuous-score AUROC values anywhere from TPR(1 − FPR) = 0.351 to 1 − FPR(1 − TPR) = 0.996 (Figure 2d); a descending uncertainty ranking of six correct predictions, 12 errors, 960 correct predictions and 22 errors reproduces the observed point exactly and has AUROC 0.351. The discriminative value of the underlying score is therefore not identified by the preserved records. Recovering it requires the per-sample scores, and the protocol for obtaining them is specified in Section 7. Second, mean prediction confidence was 0.9639 against an accuracy of 96.60%, an aggregate top-label calibration gap of −0.21 percentage points, indicating slight underconfidence on average. This is a single-bin aggregate comparison and is not an expected calibration error, which requires per-sample binning; it is reported because it is the only calibration statement the preserved records support.

What cannot be computed is stated explicitly. The full risk–coverage curve, the area under that curve (AURC), the confidence-only and entropy-only ablations, the conjunctive rule, expected calibration error, the Brier score, and error-detection AUPRC all require per-sample probability vectors that were not retained. Only two points on the risk–coverage curve are known—(coverage 1.000, selective risk 3.40%) and (coverage 0.982, selective risk 2.24%)—and the two points do not determine a curve. No attempt has been made to interpolate between them or to extrapolate beyond them. The inference pass that would produce all of these quantities requires no retraining and is specified as the first item in Section 7.

Because the rejected subset contains only 18 predictions, the stability of these proportions requires direct examination rather than assertion. The 2 × 2 partition is a sufficient statistic for the joint distribution of the two binary variables (withheld or issued; error or correct), so a non-parametric bootstrap over the 1000 predictions is fully determined by the preserved counts and can be carried out without any per-sample information. Resampling the 1000 predictions with replacement 20,000 times gives the intervals in Table 6. These intervals are conditional calculations under two assumptions the preserved records cannot verify. The first is that the 1000 images are independent observations; BRISC2025 carries no patient identifiers, so clustering by patient can be neither excluded nor adjusted for. The second is that the threshold pair was fixed independently of this partition, which Section 3.5 states was not documented. The intervals therefore describe resampling variability in the recorded counts under an unverified independent-image assumption; they are not inferential statements about a patient population, and they do not account for clustering or for threshold selection. Read on that basis, the bootstrap interval for rejection precision runs from 43.75% to 88.24% and the interval for the enrichment ratio from 17.2× to 52.8×. Within this run the withheld subset is markedly enriched for errors relative to the base rate of 3.40%, but the magnitude of that enrichment is imprecisely determined, and whether it would persist under independent threshold selection or at patient level is not established by these intervals.

**Figure 3:**
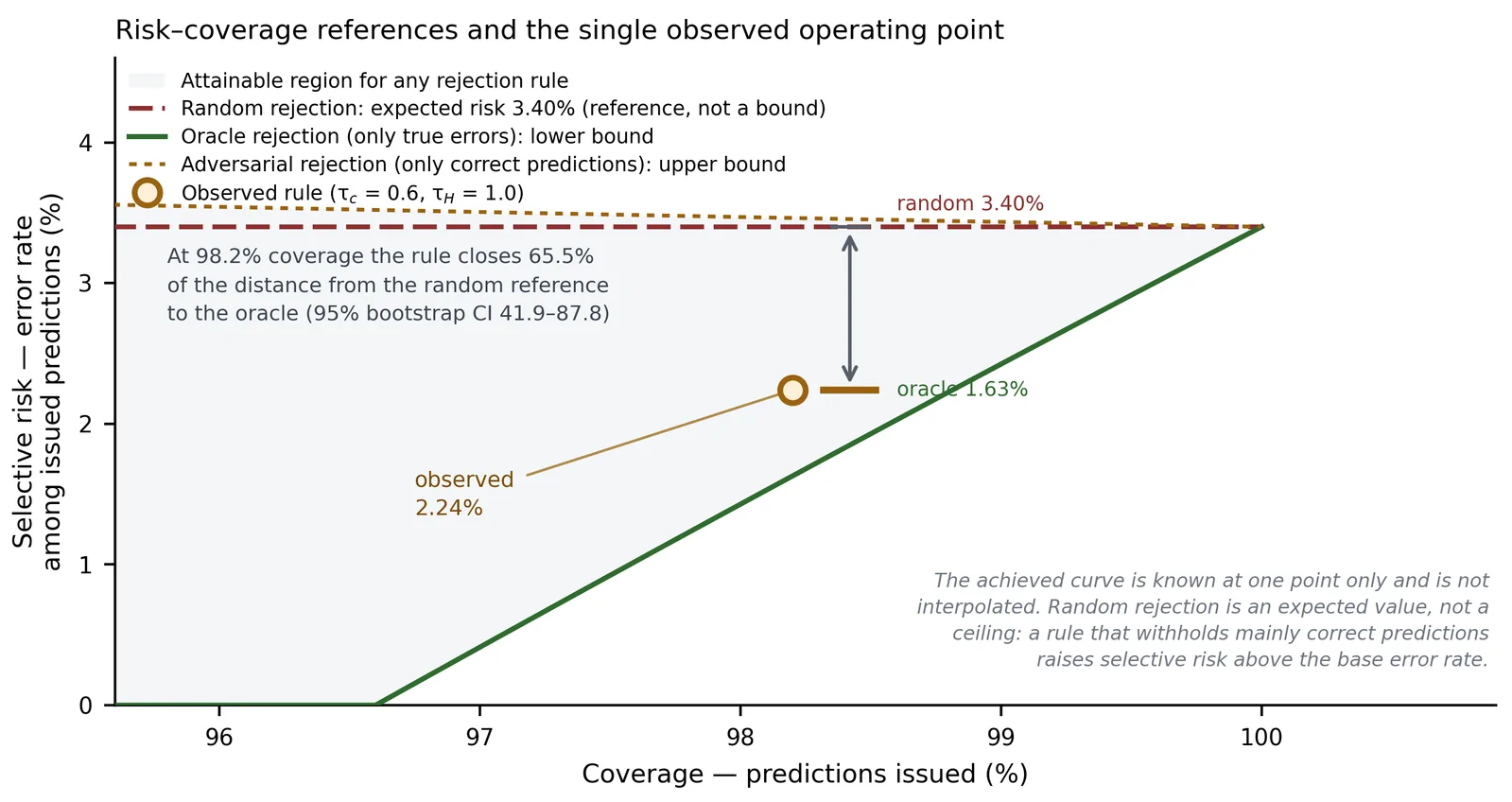
Risk–coverage references and the single observed operating point. The oracle curve, which rejects only true errors, is a true lower bound on selective risk at each coverage, and the adversarial curve, which rejects only correct predictions, is the corresponding upper bound; both are exactly determined by the 34 errors among 1000 predictions. The random-rejection line at 3.40% is the expected selective risk of a rule that carries no information about correctness. It is a reference value and not a ceiling: a rule that withholds predominantly correct predictions raises selective risk above the base error rate, so an achieved curve may lie above this line. A previous version of this figure shaded the band between the oracle and random lines as a region in which the achieved curve must lie; that description was incorrect and has been removed. The achieved curve of the disjunctive rule is known at one point only, marked at coverage 98.2%, and is not interpolated. At that coverage the rule closes 65.5% of the distance from the random reference to the oracle (95% bootstrap CI 41.9–87.8, bracket), computed under the independence assumption stated in Section 4.3. Selective risk at other coverages, the area under the achieved curve and comparisons with alternative uncertainty scores require the per-sample probability vectors, which were not preserved (Section 4.3).

Two reference points make the size of the effect interpretable, and both are exactly determined by the preserved counts. A rule that rejects 18 predictions at random carries no information about correctness and leaves selective risk at the base rate of 3.40%. An oracle that rejects 18 predictions and chooses only true errors would leave 16 errors among the 982 issued, a selective risk of 1.63%; this is the lowest value attainable at that coverage. The observed rule achieved 2.24%, which is 65.5% of the way from random rejection to the oracle (95% bootstrap CI 41.9–87.8). Figure 3 places these reference points and the single observed operating point on the risk–coverage plane. The confidence and entropy signals therefore recover about two thirds of the reduction that perfect error ranking would deliver at this coverage, which is a more informative statement of the effect size than the relative risk reduction alone.

A distinction that governs how these figures should be read is worth stating separately, because the two properties are often conflated. Selective prediction requires only that the uncertainty score rank errors above correct predictions; it does not require the scores to be calibrated probabilities. A model whose confidences are systematically too high can still be an excellent error detector, provided the ordering is informative, and a perfectly calibrated model can be a poor one if its errors are spread uniformly across the confidence range. The quantities reported in this section—sensitivity, specificity, enrichment, the AUROC bound and the oracle fraction—are all properties of the ranking and are therefore the appropriate evidence for a selective-prediction claim. They say nothing about whether the softmax outputs are calibrated in the probabilistic sense, and this manuscript makes no calibration claim beyond the aggregate gap reported above. Establishing calibration would require the reliability diagram and expected calibration error specified in Section 7.

### 4.4. Error Structure of the Vision–Language Classifier

Reviewer comment on the previous version asked for representative MRI examples of correct predictions, false positives and false negatives. The per-case prediction records for the vision–language component were not preserved: the experimental logs retain aggregate accuracy, macro-F1, per-class precision, recall and F1, the rejection rate, and confidence and entropy summary statistics, but not the image-level identifiers, predicted labels or confidence values that would be needed to locate and display an individual correct, incorrect, or withheld case. A case-level qualitative figure for this component cannot therefore be reconstructed from the surviving records, and none is presented; producing one by selecting illustrative images post hoc, without the original prediction record, would not constitute evidence about this model’s behaviour. Section 7 specifies the audit that would produce it.

The class-level error structure, in contrast, is fully determined by the preserved per-class metrics and the known class supports, and is reported in Table 7. Each class contributes a count of false negatives (reference cases of that class assigned elsewhere) and false positives (cases of other classes assigned to it); both sum to the 34 errors, which provides an internal consistency check on the table. Meningioma is the dominant source of missed cases (23 false negatives, 92.48% recall) while glioma is the dominant source of over-calls (15 false positives, 94.25% precision), a pattern consistent with the radiological similarity between these classes on T1-weighted imaging.

One consequence of this structure is worth separating out, because it concerns the error that matters most clinically. Predicted “no tumour” labels were issued 141 times; 140 of these were correct and one was a tumour-bearing image assigned to the no-tumour class. Conversely, all 140 tumour-free images were correctly identified, so no tumour-free scan was labelled as containing a tumour. At the level of tumour detection rather than tumour typing, the classifier missed 1 of 860 tumour-bearing images (0.12%, 95% CI 0.02–0.66) and raised no false alarms (0 of 140, 95% CI 0.00–2.67). The remaining 33 errors were confusions between tumour types in images correctly recognised as abnormal. This decomposition is exact: the no-tumour row and column of the confusion matrix are fully determined by the preserved marginals. The off-diagonal structure among the three tumour classes is not, and is not reported.

### 4.5. Volumetric Tumour Segmentation

Table 8 summarises the three evaluated architectures. nnU-Net (3D full-resolution) outperformed SegResNet and the standard 3D U-Net across all sub-regions and was selected as the final framework. This comparison is a controlled ablation: preprocessing and evaluation were matched, and the architecture is the only factor varied.

On the 74-case held-out cohort, the best single fold achieved a mean Dice of 0.7948 and the three-fold ensemble 0.8150 (Table 9), an absolute improvement of +0.0202 Dice. This is reported as an absolute difference in Dice points rather than as a percentage, because expressing it as a percentage invites confusion between the relative gain (2.54%) and the absolute gain in Dice points. The gain was largest in the sub-region where the single fold was weakest (NCR/NET, +0.0270) and smallest where it was strongest (ET, +0.0114), which is the pattern expected if ensembling principally reduces variance.

**Figure 4:**
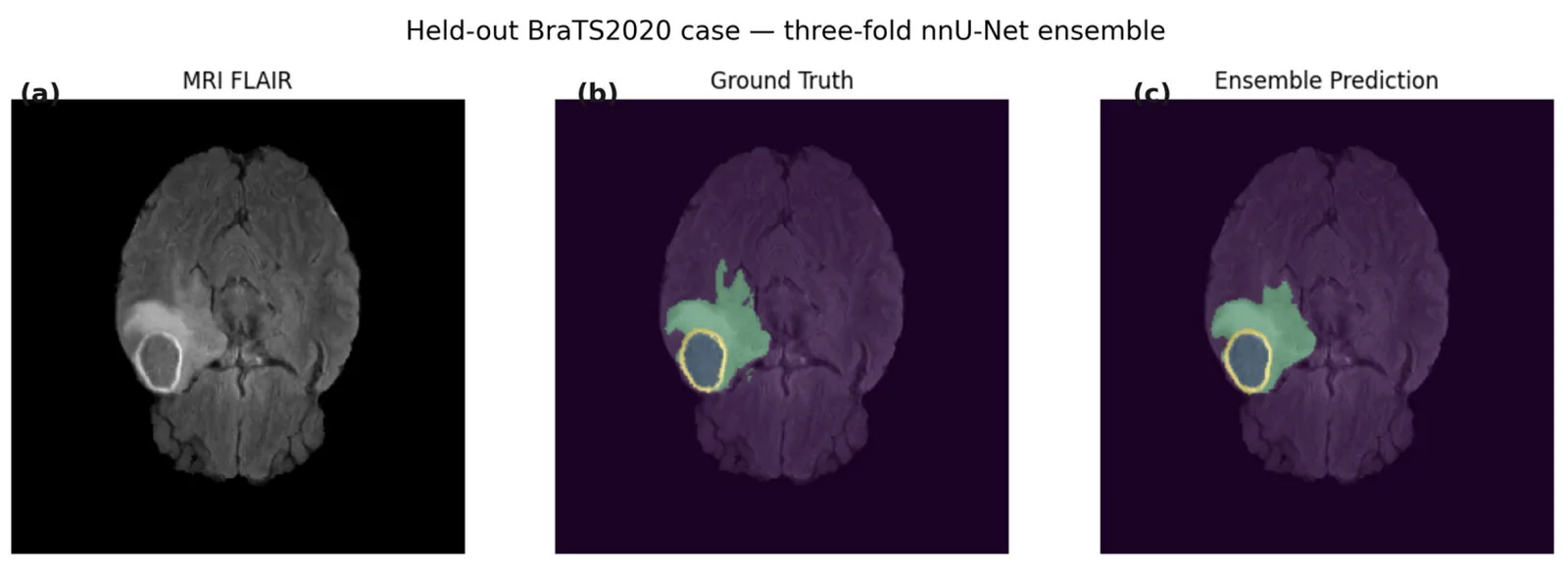
Representative case from the 74-case held-out BraTS2020 cohort. (**a**) FLAIR image; (**b**) reference annotation; and (**c**) three-fold nnU-Net ensemble prediction. Overlay colours denote the annotated sub-regions (oedema in green, enhancing tumour outlined in yellow, necrotic core in dark blue). Agreement is close for the enhancing rim and the main oedema body; the ensemble under-segments the superior extension of the oedema visible in (**b**). This panel is a single illustrative case drawn from the preserved visualisation records and supports no quantitative claim; per-case Dice values for this cohort were not preserved (Section 4.6).

Figure 4 shows a representative held-out case with the FLAIR image, the reference annotation and the three-fold ensemble prediction. The predicted sub-region boundaries follow the reference closely for the enhancing rim and the bulk of the oedema, and the visible discrepancy is a superior extension of the oedema region present in the reference that the ensemble under-segments. This is a single case selected from the preserved visualisation records and is included to make the nature of the segmentation output concrete; it is not a quantitative result and no inference is drawn from it. The distribution of per-case performance, including the weakest cases, is discussed in Section 4.6.

### 4.6. Statistical Assessment of the Ensemble Gain

The per-case Dice values underlying the ensemble result were not preserved outside the published figure. To characterise the case-level spread without fabricating values, the 74 case-level scores were recovered computationally from that figure by locating plotted marker positions through colour-matching and converting pixel coordinates to data values using the axis gridlines as calibration. The procedure reproduced the aggregate mean Dice reported above to within 0.001 (recovered mean 0.8160 against reported 0.8150). The recovered values give a descriptive mean of 0.816 with a standard deviation of 0.107, with a small number of cases scoring between 0.43 and 0.57.

These values are reported as descriptive only, and no confidence interval or inferential test is computed from them. In the previous version of this manuscript a one-sample Wilcoxon signed-rank test, a *t*-test and a bootstrap confidence interval were reported from these reconstructed values; those results have been withdrawn. Reconstructed values carry an estimated pixel-level imprecision of approximately ±0.01–0.02 Dice per case, and no equivalent per-case record exists for the single-fold evaluation, so neither a one-sample nor a paired test can be justified. The +0.0202 Dice improvement is therefore presented as a descriptive point estimate.

Because the recovery of per-case values is central to what this component can support, the attempt was repeated with two independent marker-detection procedures and the results compared against the reported aggregate. One procedure resolves 64 of the 74 plotted markers, because points that fall close together on the horizontal axis merge, and returns a mean of 0.826. A second, which forces 74 evenly spaced sampling positions across the plotted range, returns 74 values with a mean of 0.821. The reported aggregate is 0.815. The recovered mean therefore varies by roughly 0.006 to 0.011 Dice depending on implementation choices that have no principled basis for preference. The difference under examination is +0.0202 Dice, so the recovery noise is between one third and one half of the effect size. Median and interquartile values computed from such reconstructions would inherit that instability, and are not reported for that reason.

A separate and more fundamental obstacle applies to the paired comparison specifically. Recovery can in principle be attempted for the ensemble arm, because a per-case figure for it exists. No equivalent figure or record exists for the single-fold arm, so the paired differences cannot be formed at all, and neither a paired confidence interval nor a paired test can be constructed regardless of how accurately the ensemble values are recovered. Regenerating the per-case Dice values properly requires re-running inference with the stored fold checkpoints and recording per-case scores for both arms; this needs no retraining and is specified in Section 7.

This study consequently reports no inferential evidence for the ensemble difference. A difference of +0.0202 Dice across three sub-regions, consistent in direction and largest where the single fold was weakest, is suggestive but not established. Section 7 specifies the paired analysis that would settle it.

### 4.7. Glioma-Grade Classification: A Negative Result

This component is reported as a negative result. The evidence does not support a claim of usable glioma-grade discrimination on this cohort, and the demonstration of that failure, with the statistics that establish it, is more useful than the aggregate accuracy figure that conceals it.

In the architecture-selection evaluation, DenseNet121 achieved 85% accuracy against 82% for the 3D CNN and 79% for ResNet50, and was selected as the final architecture. On the separate validation partition, the confusion matrix shows 745 of 820 HGG samples and 19 of 212 LGG samples were correctly classified (Figure 4a).

Three different accuracy figures exist in our records for this model: 85% from the architecture-selection evaluation, 81% recorded as the best validation accuracy reached during training, and 74.03% implied by the validation confusion matrix. They derive from different evaluation runs and checkpoints and cannot be reconciled from the surviving records. Every claim in this section is therefore based on the 74.03% figure, because it is the only one accompanied by a confusion matrix from which class-aware statistics can be computed and independently checked; the other two are reported only to document the discrepancy, not to characterise the model. That a three-way disagreement about the accuracy of our own classifier cannot be resolved is itself part of the reason this component is reported as a negative result requiring re-execution.

Computing the full metric set directly from the confusion matrix (Table 10) shows that performance is driven almost entirely by the majority class. Balanced accuracy is 49.91%, indistinguishable from chance. Cohen’s κ is −0.0023 and MCC is −0.0026, both consistent with no better-than-chance agreement once class prevalence is accounted for. LGG recall is 8.96% with a 95% Wilson interval of 5.81–13.57%, which excludes any value compatible with reliable minority-class detection.

The comparison that most directly characterises this result is against a constant classifier that assigns every sample to HGG (Figure 4b). That baseline achieves 79.46% accuracy (95% CI 76.89–81.81) where the model achieves 74.03% (95% CI 71.27–76.61); the intervals do not overlap. A McNemar test on the paired samples gives χ^2^ = 32.18 with *p* = 1.4 × 10^−8^ (exact binomial *p* = 4.8 × 10^−9^). These quantities are reported descriptively and not as a test of significance: the paired units are multi-slice representations rather than patients, so the independence assumption of the test is violated and the *p*-value is anti-conservative by an unknown amount. What the counts establish without recourse to a test is that in this run the model is less accurate than the trivial constant-output baseline, because the 19 LGG cases it recovers are outweighed by the 75 HGG cases it loses, and that its balanced accuracy is at chance level. A patient-level reanalysis would be required before any inferential claim could be attached to the difference.

**Figure 5:**
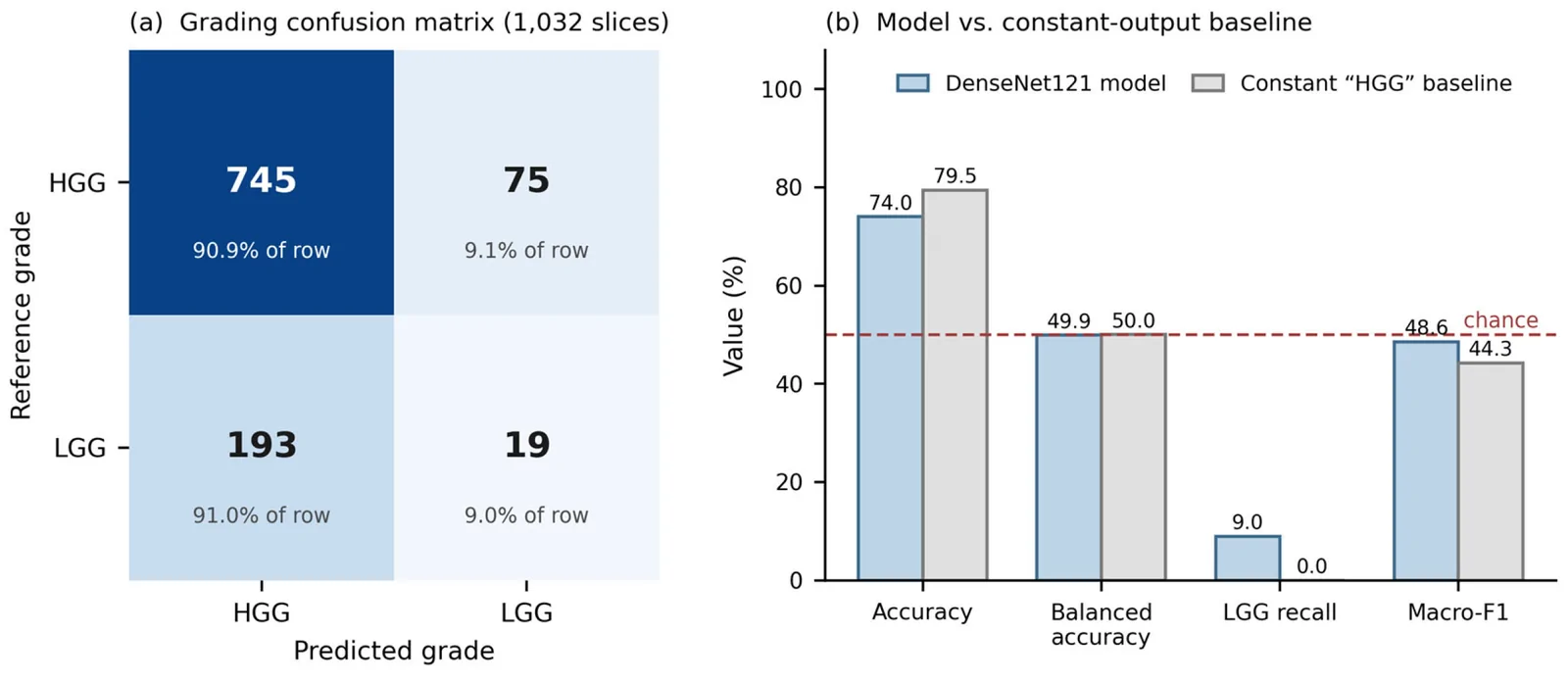
Glioma-grade classification as a negative result. (**a**) Validation confusion matrix (1032 multi-slice representations): the model assigns approximately 91% of both reference classes to HGG, so its behaviour approximates a constant-output classifier with added noise. (**b**) Model performance against a constant “HGG” classifier on the same partition. Accuracy favours the trivial baseline (74.03% versus 79.46%; McNemar χ^2^ = 32.18, *p* = 1.4 × 10^−8^, reported as a conditional and descriptive quantity only, because slice-level observations are not independent and the test is anti-conservative), balanced accuracy sits at chance (49.91%), and the model’s small macro-F1 advantage (48.59 versus 44.28) reflects only the 19 recovered LGG cases. All values are slice-level and are not generalisation estimates (Section 4.1).

Two caveats govern the interpretation of Table 10 and Figure 5. First, all counts are at the level of multi-slice representations, not patients; because multiple slices originate from each patient, these are not independent observations and the McNemar test is anti-conservative. The direction of the result is not in doubt, but the *p*-value should be read as descriptive. Second, and for the same reason, these figures may be optimistic: a slice-level partition allows slices from one patient to appear on both sides, so a patient-level partition would be expected to yield equal or worse performance, not better. Section 7 specifies the patient-level protocol required before any grading figure from this cohort is treated as a generalisation estimate.

### 4.8. Structured Report Generation

This component is reported as a scoping observation rather than as an evaluation, and it is placed last for that reason. The fine-tuned model was evaluated against reference impressions on the 141-case test split, achieving BERTScore precision 0.9424, recall 0.9605 and F1 0.9513, and ROUGE-1 0.6860, ROUGE-2 0.5324, ROUGE-L 0.6410 and ROUGE-Lsum 0.6411. These numbers are recorded for completeness and for the motivating observation that follows; taken alone they support no conclusion about generation quality, because no comparator of any kind was run.

A similarity score is interpretable only against a comparator, and none exists here: no untuned base model, no deterministic template, no retrieval baseline and no ungrounded generation baseline was evaluated, and no confidence intervals were computed. The value of 0.9513 therefore answers no question of the form “better than what?”. There is in addition positive reason to distrust it as a quality signal. It exceeds the 0.8054 reported by Voinea et al. [21] for a substantially larger Llama 3 8B model fine-tuned on 21,152 real hospital reports, and a three-billion-parameter model trained on 487 samples is unlikely to have outperformed that system. The more probable explanation is structural: the inputs here are templated structured findings and the targets are short impressions derived from the same annotations, so the task approximates a deterministic text transformation on which BERTScore is known to saturate. We report the comparison in order to argue against our own number rather than in support of it.

This section accordingly reports the automatic metrics as recorded and draws no conclusion from them. No claim is made that the fine-tuned model generates clinically coherent impressions, that structured-finding grounding improves output quality, or that these scores compare favourably with published systems. The baselines required to support any such claim are specified in Section 7.

During development one recurring hallucination mode was identified: the model occasionally generated physical tumour measurements in millimetres or centimetres although the upstream 2D segmentation pipeline produced only pixel-based measurements, so such measurements were not supported by the available evidence. This failure has an identifiable mechanical cause—the 2D pipeline processes PNG/JPG slices, which carry no voxel spacing—and the volumetric pipeline does not share it, since NIfTI volumes carry voxel dimensions from which physical measurements are genuinely derivable. That yields a testable prediction: unsupported physical measurements should occur at a substantially lower rate in reports grounded in volumetric findings than in reports grounded in 2D findings. Insufficient grounding to input evidence is identified as a persistent failure mode across the hallucination literature [29,30], including in constrained generation settings.

This observation is qualitative. The hallucination mode was identified during informal developmental review rather than by systematic annotation, and no rate, denominator or severity distribution is reported, because none was measured. The quantitative audit required to test the mechanism proposed above is specified in Section 7.

### 4.9. Robustness

Under brightness and rotation perturbation, 39 of 40 predictions were stable, a stability of 97.50% with a 95% Wilson interval of 87.12–99.56%. The scale of this evaluation is stated explicitly: the 40 observations comprise 10 base images under four perturbation conditions, so the number of independent units is 10, and the four observations from each image are correlated. The previous version of this manuscript reported the 97.5% figure in the Abstract without its interval or its sample size; it has been removed from the Abstract’s claims and is reported here as a pilot observation.

At this scale no robustness conclusion is drawn. The interval spans more than twelve percentage points, and four synthetic perturbations on ten images do not approach the range of real acquisition variability. The scaled protocol is specified in Section 7.

## 5. Discussion

### 5.1. Principal Finding: A Reduction in Selective Risk from Selective Prediction

For this model, this threshold pair and this partition, the observed counts are consistent with the hypothesis stated in Section 1: the rule produced a measurable reduction in selective risk at a low rejection rate. The statement is confined to what was observed here and is not a general claim about the method, because the thresholds were not selected on an independent partition and no second partition was evaluated. Withholding 1.80% of predictions removed 35.3% of all errors and reduced selective risk by 34.1% in relative terms, and the withheld predictions were enriched for errors by a factor of approximately 30 (Fisher exact *p* = 4.6 × 10^−15^, computed under the independence assumption stated in Section 4.3). The direction and size of this enrichment is what makes the observation informative. Any rejection rule raises accuracy among retained predictions if it rejects enough of them. A rule is informative only if the predictions it discards are disproportionately those that would have been incorrect, and that is what the enrichment describes. Two thirds of withheld predictions here were errors, against 2.24% of issued ones, which indicates that in this run the confidence and entropy signals carried information about correctness rather than merely about class prior.

The result extends to a vision–language classification setting the principle Fidon et al. [12] demonstrated for segmentation: an explicit abstention mechanism can improve the reliability of a system’s issued outputs without a punitive cost in throughput. It does so here using only quantities available from a single forward pass, without the ensemble machinery that stronger uncertainty estimators require [13], which substantially reduces the computational cost of obtaining the uncertainty estimate. Whether that cost profile is compatible with a particular clinical service is an implementation question this study does not address.

### 5.2. Why Coverage–Risk Matters in Medical AI

Coverage and risk trade against each other, and the rate at which one is exchanged for the other is the quantity a clinical service must evaluate; this is why the two are reported jointly. At the operating point evaluated here, 1.8 percentage points of coverage bought a 1.16 percentage point absolute reduction in error rate among issued predictions. Whether that exchange is worthwhile is not a statistical question but an operational one, and it depends on what happens to a withheld case. If an abstention routes the study to a radiologist who would have reviewed it in any case, the incremental coverage cost is negligible. If it triggers a repeat acquisition, the cost is substantially higher and the same reduction in selective risk may not justify it. Reporting the operating point without the coverage figure removes the reader’s ability to make that judgement at all, which is why the two are reported jointly throughout this manuscript and why the denominator of every accuracy figure is stated.

The same logic explains why a single operating point is not a sufficient characterisation, and this is the clearest methodological shortcoming of the present analysis. The thresholds τ_c_ = 0.6 and τ_H_ = 1.0 were set empirically, and the partition on which they were chosen is undocumented, so the reported point may be optimistically placed, and the size of any such bias cannot be estimated from the available records. Two distinct deficiencies follow, and they require different remedies. The first is that behaviour is known at one threshold rather than across the range; the full risk–coverage curve would remove that dependence, and it requires only stored per-sample softmax vectors from a single inference pass. The second is the selection bias, and recovering the curve does not address it: recomputing the curve on the partition already inspected here would describe that partition more completely without making the threshold choice independent of it. Removing the selection bias requires a threshold-selection partition independent of both model fitting and the final evaluation, as specified in Section 7. Until both are supplied, the reported reduction cannot be shown to be robust to the choice of threshold, and the finding should be read as a description of how abstention concentrated errors in this run rather than as a recommendation of these particular threshold values.

### 5.3. Relationship to the Segmentation Component

The segmentation results are consistent with the ensembling literature but are weaker evidence than they appear. The ensemble improved held-out mean Dice by +0.0202 and the gain was concentrated in the sub-region where the single fold was weakest, which is the signature of variance reduction. No inferential test supports the difference, because the per-case values required for a paired comparison were not preserved, and the tests previously computed from figure-reconstructed values have been withdrawn (Section 4.6). The mean Dice reported here is also computed over raw label channels rather than the BraTS ET/TC/WT composites, so the comparisons with published BraTS results made in the previous version of this manuscript were not valid and have been removed pending recomputation.

One observation from this component does bear on the study’s central theme. Internal fold performance (EMA Dice 0.8511) exceeded held-out performance (0.7948) by a substantial margin within a single collection, which illustrates that cross-validation metrics overstate generalisation even in the absence of any domain shift. It does not test cross-institutional generalisation, which no experiment here addresses. The gap is a further instance of the study’s general point: a performance figure acquires meaning only from the evaluation protocol that produced it.

### 5.4. The Negative Finding in Glioma-Grade Classification

The grading result is negative and is reported as such. In this run the classifier was less accurate than a constant-output baseline, 74.03% against 79.46%, with chance-level balanced accuracy and near-zero κ and MCC. The McNemar statistic associated with that difference is reported in Section 4.7 as a descriptive quantity only: the comparison is made across multi-slice representations rather than patients, so the independence assumption of the test is violated and its *p*-value is anti-conservative. No claim of statistical significance is made here, and the findings that stand are the observed accuracy difference and the chance-level balanced accuracy, both descriptive of this run. The proximate cause is the pronounced class imbalance in the BraTS2020 cohort combined with the visual similarity of glioma grades on conventional MRI, a pattern consistent with broader evidence that imbalance biases medical imaging classifiers towards the majority class [18] and with Wen et al.’s finding that grading, not segmentation, is the harder sub-problem in joint frameworks [17]. It is compounded by a slice-level partition that renders the figures non-independent and probably optimistic.

The finding is scientifically informative for two reasons. The first is corrective: a study containing many grading papers that report accuracies above 95% benefits from a controlled demonstration that a standard architecture fails on this task under honest evaluation, and that a 74.03% accuracy figure—which would not look anomalous in isolation—can coexist with balanced accuracy at chance and negative chance-corrected agreement. The second bears directly on the study’s central question. The selective-prediction result of Section 4.2 shows that a model’s confidence can carry usable information about its own correctness; the grading result shows that a model’s aggregate accuracy carries almost none about its discriminative ability when classes are imbalanced. Reliability and accuracy are separable properties, and they are separable in both directions.

It also illustrates the value of the component-wise design. An end-to-end accuracy figure would have absorbed this failure into a system-level number and concealed which stage was responsible. Evaluating the stages separately is what made the failure visible, and it is the reason the design is defended here as appropriate to the study’s aims rather than merely as a limitation of them.

### 5.5. Interpretation and Limitations of Report Generation

The report-generation results are the weakest in the study, not because the numbers are low but because they are uninterpretable without baselines. The conclusion drawn in the previous version—that structured-finding grounding produces clinically coherent impressions—has been withdrawn, because no ungrounded configuration was run for comparison and because a BERTScore F1 above that reported for a much larger model trained on far more real clinical data [21] is more plausibly explained by template overlap than by superiority. BERTScore and ROUGE measure agreement with a reference string. They do not measure factual correctness, clinical acceptability or the rate of clinically serious errors, and on short, templated reference text they saturate. They are reported here as automatic similarity measures and carry no implication of clinical validity. This component is therefore exploratory and scoping in character rather than evaluative, and it is labelled as such throughout.

This is the component of the framework where an unflagged error would reach a reader in natural language, with the fluency of the output providing no signal about its reliability. The observed hallucination mode—physical measurements generated from a pipeline that produced only pixel-based measurements—is a concrete instance: the fabricated quantity was indistinguishable in form from a supported one. Validating this component therefore requires expert review rather than more automatic metrics, and the acceptance-rate and ranking methodology established for generative report drafting in other imaging modalities [31] provides an appropriate template. Until that is done, no claim about the clinical adequacy of these outputs is warranted.

### 5.6. Stochastic Variability and Reproducibility

Every figure in this manuscript comes from a single training run with unrecorded seeds. The practical consequence is that the reported values are point estimates whose run-to-run spread is unknown, and comparisons smaller than that unknown spread cannot be resolved. This bears unequally on the three quantitative components. The selective-prediction result is the least affected, for a reason that rests on the counts rather than on any test: it is an analysis applied post hoc to the stored outputs of one fixed model, so the partition of those outputs into withheld and issued predictions, and of errors within each, is exact for that model and does not vary between runs. No appeal is made here to the magnitude of any *p*-value, since the tests reported in Section 4.3 are conditional on an unverified independence assumption and on a threshold pair chosen without an independent partition. What repeated seeds would establish is whether a similarly trained classifier reliably exhibits the same enrichment, not whether this one did.

The segmentation comparison is more exposed. A +0.0202 Dice difference is well within the range that seed variation alone can produce in volumetric segmentation, and without repeated runs the ensemble difference cannot be separated from initialisation noise; this is an additional reason, beyond the missing per-case values, for reporting it descriptively. The grading result is exposed in a different way: the reported accuracy is a single-run point estimate, and the three irreconcilable accuracy values in our records (Section 4.7) are themselves an illustration of how unstable single-configuration reporting can be. Because the seeds were not recorded and the configuration was not rerun, the run-to-run distribution of this result is unknown. A previous version of this manuscript argued that a model at chance-level balanced accuracy would not become a useful classifier under a different seed; that inference cannot be drawn from a single run with unrecorded seeds and has been withdrawn. No statement is made here about what a different seed would or would not produce.

Evidence from other domains supports treating this as a first-order methodological requirement rather than a formality. In automated fault detection and diagnosis for building air-handling units, self-supervised and semi-supervised time-series learning methods are evaluated over repeated randomised runs with dispersion reported alongside central tendency, because single-run comparisons in that setting have been shown to be unstable [27,28]. That work is not evidence about brain MRI, and no numerical transfer is implied; it is cited as empirical support for the general claim that single-configuration deep learning results are a weak basis for comparison. The repeated-seed protocol specified in Section 7—three seeds per configuration, with mean ± standard deviation and coefficient of variation reported for every primary metric—is the appropriate remedy, and it was not performed in this study.

### 5.7. Reliability, Hallucination Control and Data Security as Distinct Requirements

A reviewer of the previous version observed that trustworthy medical imaging AI is often discussed as though it were a single property, when it is in fact several. The distinction matters for stating what this study does and does not address. Model reliability—whether a prediction is likely to be correct—and uncertainty estimation—whether the model can signal that likelihood—are the properties examined here, and hallucination control, the problem of generated text asserting content the input does not support, is examined qualitatively in Section 4.8. These are properties of the model’s behaviour given data it has legitimately received.

Confidentiality, integrity and availability of the imaging data itself are a separate axis and are not addressed by any experiment reported here. MRI volumes are large, highly identifiable and frequently transmitted between acquisition, archive and analysis systems, and protecting them in storage, in transit and during processing is an active research area in its own right, encompassing chaos-based and other encryption schemes designed for the statistical properties of medical images [32] as well as the cryptanalysis that establishes whether such schemes actually resist attack [33]. The latter literature is a useful methodological parallel to the present study: a cryptosystem that reports favourable histogram and entropy statistics may still fall to a chosen-plaintext attack, just as a classifier that reports favourable accuracy may still fail to identify its own errors. In both settings the summary statistic is not the property that matters.

This study addresses none of the security dimensions. It used publicly available, de-identified collections, performed no transmission or storage of identifiable patient data, and implemented no encryption, access-control or integrity-verification mechanism. Data security is therefore outside the scope of this manuscript and is not claimed as a contribution. A deployed system built on components of this kind would require both the reliability mechanisms examined here and the confidentiality and integrity guarantees examined in that literature; neither substitutes for the other, and the present work supplies evidence about the first only.

### 5.8. Clinical Interpretation and the Boundaries of This Study

The overall message is that high predictive accuracy alone is not sufficient evidence that a model can identify its own errors, and that this capability can differ substantially between components of one framework. The vision–language classifier was both accurate and able to identify a meaningful share of its own errors. The grading classifier reached 74.03% accuracy while discriminating at chance. A system built from both, and assessed by a single aggregate figure, would have reported a number that describes neither. Explicit mechanisms that identify uncertain cases, evaluated per component and reported with coverage, are therefore not an optional refinement on top of accuracy reporting but a separate requirement.

The boundaries of what has been shown should be equally clear. These are component-wise results on public collections. No experiment passed a set of patient cases through the framework end to end; no evaluation used data from outside the collection that supplied the corresponding training data; no practising radiologist assessed any output under a blinded protocol; and the reliability behaviour reported in Section 4.2 was measured on 1000 two-dimensional slices from one dataset, not on a clinical population. Nothing here establishes that this framework, or any component of it, is ready for clinical use, and no such claim is made. The results support a methodological conclusion about how reliability should be measured and reported, not a clinical conclusion about what should be deployed.

### 5.9. Requirements for Validation

Three requirements follow directly from the design of this study and would have to be met before any clinical interpretation could be attempted. The first is the complete uncertainty characterisation described in Section 5.2—the risk–coverage curve, a rejection-rule ablation and a calibration analysis—which converts the present single-point demonstration into a characterisation of the method. The second is a patient-matched end-to-end evaluation in which the same cases traverse segmentation, structured-finding extraction, report generation and selective prediction in sequence, so that error propagation between stages can be measured rather than assumed absent. The third is external validation on a cohort from a different institution, together with expert review of generated reports under a blinded protocol. Section 7 specifies each of these as a concrete protocol with its required inputs.

It is also worth stating which comparisons in this study function as genuine controlled ablations, since the strength of an inference depends on it. Table 8 isolates the contribution of the nnU-Net architecture against SegResNet and a 3D U-Net under matched preprocessing. Table 4 isolates the contribution of the rejection mechanism by holding the underlying classifier fixed and varying only whether rejection is applied. These two support direct attribution. The ensemble difference in Table 9 does not, since it combines three folds simultaneously rather than removing one at a time, and the report-generation results in Section 4.8 do not, since no alternative configuration was run. The distinction is flagged rather than allowing the strength of the two genuine ablations to be read onto the two comparisons that are not controlled in the same way.

## 6. Limitations

No external validation. Every evaluation cohort originates from the same collection as its corresponding training data. The 74-case segmentation cohort is an official held-out split of the pooled BraTS2020 collection, not a separate site, scanner or population. No result here constitutes external or cross-institutional validation.

No end-to-end validation. The four components were evaluated on distinct cohorts and never as a single patient-matched pipeline. No case-level end-to-end metric is reported because no such experiment was performed, and Figure 1 is a conceptual architecture rather than a validated system.

No clinical validation. No practising radiologist independently evaluated any system output under a blinded protocol. The developmental review described in Section 3.8 was informal and conducted by the research team. Claims of clinical adequacy, readability or diagnostic value are not made anywhere in this manuscript.

Single configuration and unrecorded seeds. Each component was trained and evaluated under a single random partition and a single initialisation, with seeds and library versions unrecorded. The reported figures reflect one experimental configuration rather than a distribution over seeds and splits, so their stability cannot be assessed from the evidence presented. Repeated-seed evaluation with mean ± standard deviation reporting was not performed. This is a material limitation for the segmentation and grading components in particular (Section 5.6).

Slice-level evaluation for grading. The grading partition allows slices from one patient to fall on both sides. Those figures are not generalisation estimates and are not presented as such.

Unvalidated thresholds and a single operating point. The selective-prediction thresholds were chosen empirically, the partition on which they were chosen is not documented, and no sensitivity analysis has been performed. If they were selected on the test partition, Table 4 is optimistically biased. Table 4 also reports one threshold pair rather than the risk–coverage curve, so the reported reduction cannot be shown to be robust to the choice of threshold.

No risk–coverage curve, rule ablation or calibration error. The per-sample probability vectors were not retained, so the full risk–coverage curve, the area under it, the confidence-only and entropy-only ablations, the conjunctive rule, expected calibration error, the Brier score and error-detection AUPRC could not be computed (Section 4.3). Only the trivial full-coverage point and the single realised operating point on the achieved curve are known. The AUROC of the underlying continuous uncertainty score is not identified by the preserved records: [0.351, 0.996] is the range of values logically consistent with the observed operating point, and no narrower statement is available from these data. The principal selective-prediction finding is therefore reported at a single empirically chosen operating point as exploratory evidence, not as an established reliability result.

No case-level qualitative error analysis for the vision–language component. The per-case prediction records were not preserved, so representative correct, incorrect and withheld cases cannot be identified from the surviving data (Section 4.4). The class-level error decomposition in Table 7 is derivable from the preserved metrics, but the image-level examples a reader would need in order to inspect failure modes visually are not.

No patient-level independence for the vision–language cohort. BRISC2025 is distributed as labelled images without patient identifiers, so it cannot be established whether multiple images originate from the same patient. The unit of analysis is therefore the image, aggregation to patient level is not possible, and the Wilson intervals reported in Section 4.2 and Section 4.3 treat images as independent observations. If images from one patient appear in the partition more than once, those intervals are narrower than the data warrant and the generalisation of the reliability finding to a patient population is correspondingly weaker. No claim of patient-level independence is made anywhere in this manuscript.

Narrow robustness evaluation. The perturbation evaluation covers 10 images and 4 synthetic conditions, and does not approach the range of real-world acquisition variability.

Incomplete experimental records. Several implementation details were not preserved and cannot be reported: the learning rate of the report-generation fine-tuning run; the slice count, slice-selection criterion and case-level aggregation rule for glioma grading; the partition on which the rejection thresholds were selected; per-case Dice values for the segmentation cohort; per-case predictions for the vision–language cohort; and random seeds and library versions for all components. The grading component in particular is not independently reproducible from the description given here. These absences are reported explicitly rather than as a general caveat, and Section 7 specifies the re-execution required to remove them.

Possible cohort overlap between components. The BraTS-GLI subset of RadGenome-Brain and the BraTS2020 collection derive from related BraTS cohorts, and patient-level overlap between the report-generation and segmentation sets cannot be excluded from available records.

No baselines and no expert evaluation for report generation. The automatic similarity scores are reported without confidence intervals, without an ungrounded comparator, without an untuned-model or template baseline, and without blinded expert review. No annotation of factual errors, unsupported measurements, clinically important omissions or serious-error rate was performed. BERTScore and ROUGE are therefore reported as automatic similarity measures only and establish nothing about clinical correctness or acceptability.

## 7. Future Work

The analyses required to complete this study are listed below in the order in which they are intended to be carried out, with the required inputs stated so that each is directly reproducible. The first three address the primary scientific question and its principal weaknesses; the remainder address the complementary components.

- Complete uncertainty characterisation under a clean threshold-selection protocol. This is the principal outstanding analysis and the one that would convert the present exploratory finding into a characterisation of the method. The design has four requirements. First, the thresholds must be fixed on data independent of both model fitting and the final evaluation; because the 1000-image BRISC2025 test partition has already been inspected in the present study, it can no longer serve as an untouched evaluation set, and a fresh inference pass over it would not repair that. Either a validation split must be carved from data not used for LoRA fine-tuning and not previously examined, with a genuinely held-out partition reserved for evaluation, or, if no such partition can be constructed from the available collections, the evaluation design must state that limitation explicitly and the resulting figures must be reported as internal rather than held-out. Second, checkpoint and partition provenance must be documented: the adapter checkpoint hash, the exact partition membership, the random seeds and the inference configuration must be recorded before the evaluation is run. Third, the per-image outputs must be retained—predicted label, reference label, full softmax vector, derived confidence and predictive entropy—so that every downstream quantity is recomputable without rerunning inference. Fourth, the reporting must cover the risk–coverage curve across the full-coverage range and its area (AURC); the rule ablation comparing confidence only, entropy only, the disjunction and the conjunction; the error-detection summary as AUROC and AUPRC of the continuous uncertainty score with confidence intervals, which the present records cannot identify; and the calibration summary as a reliability diagram with expected calibration error and Brier score. No retraining is required at any stage; the analysis needs one inference pass and the record-keeping described above. Until it is completed, the selective-prediction result in this manuscript remains a description of one operating point.
- Case-level qualitative error audit for the vision–language classifier. Re-running inference with per-image logging of the predicted label, reference label, confidence and entropy would permit the representative correct, false-positive, false-negative and withheld cases requested by reviewers to be identified from the record rather than selected post hoc, and would also yield the full 4 × 4 confusion matrix that the preserved marginals underdetermine.
- Repeated seeds and partitions across all components, with at least three seeds per configuration, reporting mean ± standard deviation and coefficient of variation for every primary metric rather than single-configuration point estimates.
- Per-case segmentation logs and BraTS-convention recomputation. Recovering or regenerating per-case Dice enables the paired Wilcoxon and bootstrap comparison, fold-to-fold variability estimation, and recomputation under the ET/TC/WT convention required for literature comparison.
- Patient-level grading re-run. Grouped five-fold cross-validation on patient identifiers, three seeds, three architectures, with an explicit majority-class baseline, reporting balanced accuracy, macro-F1, MCC and κ as mean ± SD across folds.
- Class-imbalance ablation for grading. Baseline against class-weighted cross-entropy (inverse-frequency weighting, HGG:LGG ≈ 1:3.9), focal loss with γ = 2, and LGG oversampling to a balanced training ratio, evaluated independently and in combination under the patient-level protocol, with balanced accuracy and macro-F1 as primary outcomes.
- Report-generation baseline suite, hallucination audit and blinded expert evaluation. Four comparators are required—an untuned base model, a deterministic template, a retrieval baseline and an ungrounded generation baseline—each reported with confidence intervals. These would be followed by systematic annotation of unsupported statements with an explicit denominator, and then by blinded review of a random sample of generated impressions by at least two practising radiologists, scoring factual correctness, clinical acceptability, unsupported measurements or conclusions, and clinically important omissions, with a serious-error rate as the primary safety outcome and an acceptance-rate and ranking methodology of the kind established for generative report drafting in other imaging modalities [31].
- Robustness at scale, extending the perturbation protocol of Section 4.9 to the full test partition and to acquisition-realistic perturbations rather than four synthetic conditions on ten images.
- External validation. An independent cohort with compatible modalities and labels will be sought; UCSF-PDGM is the leading candidate, since it originates from a different institution and carries WHO-grade information supporting both segmentation and grading validation. Any cohort drawn from the BraTS collections must first be de-duplicated against BraTS2020 at case level; BraTS2021 in particular contains the BraTS2020 cases and cannot be used as an independent cohort without that exclusion.
- Case-level end-to-end evaluation. Passing the 74 held-out cases through segmentation, structured-finding extraction, report generation and selective prediction in sequence would produce the end-to-end metrics this study does not report, and would allow error propagation between stages to be measured.

## 8. Conclusions

This study asked whether uncertainty-aware selective prediction improves the reliability of a biomedical vision–language classifier by concentrating errors among withheld cases, and at what coverage cost. The evidence assembled here is a single-operating-point case study, and it answers that question descriptively rather than conclusively. Withholding 1.80% of predictions removed 35.3% of all errors and reduced selective risk from 3.40% to 2.24%, a relative reduction of 34.1%, with withheld predictions enriched for errors by a factor of approximately 30; the rule withheld these errors while issuing 99.38% of the predictions that were correct, and at the realised coverage it closed 65.5% of the distance from a random reference to an oracle that rejects only true errors. Within this run the reduction in selective risk was therefore large relative to its coverage cost, and it arose because confidence and entropy carried information about correctness rather than because predictions were discarded indiscriminately. What the study does not establish is that this behaviour is a property of the method rather than of this threshold pair on this partition. The thresholds were chosen empirically on an undocumented partition; the per-sample probability vectors were not preserved, so the risk–coverage curve, its area, the confidence-only and entropy-only comparisons and a calibration analysis could not be computed; the evaluation is at image level on one public collection that carries no patient identifiers. The selective-prediction result is therefore reported as exploratory evidence, and the validation experiment that would convert it into a demonstration is specified in Section 7.

The complementary components qualify that conclusion in a way that is central to its interpretation. Three-fold nnU-Net ensembling improved held-out mean Dice by +0.0202, reported descriptively because the per-case data required for a paired test were not preserved; it provides supporting evidence for volumetric tumour characterisation but no inferential evidence for the ensemble difference itself. Volumetric glioma grading failed: in this run it was less accurate than a constant-output baseline, with chance-level balanced accuracy and near-zero chance-corrected agreement, and it is reported as a negative result without an accompanying significance claim, since the slice-level units are not independent. Structured report generation produced high automatic similarity scores that are not interpretable without baselines and expert review, and no claim of clinical adequacy is made for it.

The general conclusion the study supports is that high predictive accuracy is not by itself evidence that a model can identify its own errors, and that different components of one analysis framework can differ substantially in this respect: one classifier here identified a meaningful fraction of its own errors, while another reached a respectable-looking accuracy with no usable discrimination at all. Reliability therefore has to be measured explicitly, per component, with coverage reported alongside risk.

These results are component-wise. They do not constitute an end-to-end validated pipeline, they were not validated on data external to their source collections, they rest on single training runs with unrecorded seeds, and they were not evaluated by independent clinical experts. The analyses required to address each of these gaps are specified in Section 7.

## Figures and Tables

**Table 2:** Report-generation fine-tuning configuration.

Setting	Value
Base model	Meta Llama 3.2 3B Instruct
Quantisation	4-bit NormalFloat (NF4), BitsAndBytes
Fine-tuning method	QLoRA via PEFT
LoRA rank (r)	16
LoRA alpha	32
LoRA dropout	0.05
Training epochs	2
Maximum sequence length	1024 tokens
Dataset (filtered RadGenome-Brain)	697 cases (487 train/69 validation/141 test)
Hardware	Single NVIDIA Tesla T4 (16 GB)
Learning rate	Not preserved (Section 6)

**Table 3:** Per-class performance of the vision–language classifier prior to rejection, with 95% Wilson score intervals (n = 1000).

Class	Recall (95% CI)	Precision (95% CI)	Specificity (95% CI)
Glioma	96.85% [93.91, 98.40]	94.25% [90.74, 96.49]	97.99% [96.71, 98.78]
Meningioma	92.48% [88.97, 94.94]	97.25% [94.67, 98.60]	98.85% [97.74, 99.41]
Pituitary	99.00% [97.10, 99.66]	96.74% [94.11, 98.22]	98.57% [97.39, 99.22]
No tumour	100.00% [97.33, 100.00]	99.29% [96.09, 99.87]	99.88% [99.34, 99.98]

**Table 4:** Selective-prediction behaviour at the operating threshold (τ_c_ = 0.6, τ_H_ = 1.0), n = 1000.

Quantity	Value	95% CI	Note
Coverage	98.2% (982/1000)	[97.17, 98.86]	Predictions issued
Rejection rate	1.80% (18/1000)	[1.14, 2.83]	Predictions withheld as “unknown”
Accuracy before rejection	96.60% (966/1000)	[95.29, 97.56]	All predictions issued
Selective accuracy	97.76% (960/982)	[96.63, 98.52]	Accuracy among issued
Baseline risk	3.40% (34/1000)	[2.44, 4.71]	Error rate, no rejection
Selective risk	2.24% (22/982)	[1.48, 3.37]	Error rate after rejection
Absolute risk reduction	1.16 percentage points	—	3.40% → 2.24%
Relative risk reduction	34.1%	—	—
Error-capture rate	35.3% (12 of 34)	[21.49, 52.09]	Errors withheld
Rejection precision	66.7% (12 of 18)	[43.75, 83.72]	Withheld that were errors
Rejection enrichment	66.7% vs. 2.24% (≈30×)	OR 87.3	Fisher exact *p* = 4.6 × 10^−15^

**Table 5:** Operating characteristics of the rejection rule considered as a detector of its own errors, computed from the 2 × 2 partition shown in Figure 2a. All quantities are exactly determined by the preserved records; no per-sample data were used or required.

Quantity	Value	95% CI	Note
Errors withheld (true positives)	12 of 34	—	From Figure 2a
Correct predictions withheld (false positives)	6 of 966	—	From Figure 2a
Sensitivity (=error-capture rate)	35.29%	[21.49, 52.09]	Errors that were withheld
Specificity	99.38%	[98.65, 99.72]	Correct predictions that were issued
Precision (=rejection precision)	66.67%	[43.75, 83.72]	Withheld predictions that were errors
Negative predictive value (=selective accuracy)	97.76%	[96.63, 98.52]	Issued predictions that were correct
Youden’s J	0.347	—	Sensitivity + specificity − 1
Balanced accuracy of the detector	67.34%	—	Mean of sensitivity and specificity
Precision lift over the base error rate	19.6×	—	66.67% against a 3.40% base rate
Diagnostic odds ratio	87.3	—	Fisher exact *p* = 4.6 × 10^−15^
AUROC of the underlying uncertainty score	not identified	—	Not a sampling interval: [0.351, 0.996] is the range of values logically consistent with the observed operating point (Section 4.3, Figure 2d)
Aggregate top-label calibration gap	−0.21 pp	—	Mean confidence 0.9639 vs. accuracy 96.60%; not ECE

**Table 6:** Stability of the selective-prediction result. Wilson intervals are shown alongside non-parametric bootstrap intervals from 20,000 resamples of the 1000 predictions, together with the random-rejection and oracle reference points at the realised coverage of 98.2%. All values follow from the preserved 2 × 2 partition; no per-sample information was used.

Quantity	Point Estimate	95% Wilson CI	95% Bootstrap CI
Rejection precision (withheld that were errors)	66.67% (12/18)	[43.75, 83.72]	[43.75, 88.24]
Error-capture rate	35.29% (12/34)	[21.49, 52.09]	[19.23, 51.85]
Selective risk	2.24% (22/982)	[1.48, 3.37]	[1.33, 3.19]
Rejection enrichment (withheld ÷ issued error rate)	29.8×	—	[17.2, 52.8]
Selective risk—random rejection at the same coverage	3.40%	—	reference
Selective risk—oracle rejection at the same coverage	1.63% (16/982)	—	reference
Fraction of the oracle reduction realised	65.5%	—	[41.9, 87.8]

**Table 7:** Class-level error structure of the vision–language classifier before rejection, derived from the per-class metrics of Table 3 and the class supports of the 1000-image test partition. FN denotes reference cases of the class assigned to another class; FP denotes cases of other classes assigned to this class.

Class	Reference Cases	Correct	Predicted as This Class	FN	FP	Net
Glioma	254	246	261	8	15	+7
Meningioma	306	283	291	23	8	−15
Pituitary	300	297	307	3	10	+7
No tumour	140	140	141	0	1	+1
Total	1000	966	1000	34	34	0

**Table 8:** Comparison of 3D segmentation architectures and per-fold nnU-Net internal validation performance on BraTS2020.

Architecture/Fold	EMA val. Dice	NCR/NET	ED	ET
SegResNet	0.7538	0.7100	0.7700	0.7800
3D U-Net	0.7300	0.6900	0.7400	0.7600
nnU-Net Fold 1 (best)	0.8511	0.8236	0.8523	0.8841
nnU-Net Fold 2	0.8269	0.7927	0.8451	0.8952
nnU-Net Fold 3	0.8356	0.7788	0.8638	0.9021

**Table 9:** Single-fold versus three-fold ensemble performance on 74 held-out validation cases, under the NCR/NET–ED–ET convention defined in Section 3.9. Values under the BraTS ET/TC/WT convention are required for comparison with published BraTS results and are pending recomputation.

Tumour Region	Fold 1 (Single)	Ensemble (Folds 1–3)	Absolute Difference
NCR/NET	0.7221	0.7491	+0.0270
ED	0.8261	0.8483	+0.0222
ET	0.8361	0.8475	+0.0114
Overall Mean Dice	0.7948	0.8150	+0.0202

**Table 10:** Complete metric set for glioma-grade classification, computed directly from the validation confusion matrix (745/820 HGG and 19/212 LGG correct). All values are slice-level.

Metric	Value	95% CI	Interpretation
Accuracy	74.03% (764/1032)	[71.27, 76.61]	Below baseline
Majority-class baseline	79.46% (820/1032)	[76.89, 81.81]	Constant “HGG” classifier
Balanced accuracy	49.91%	—	Chance level
Macro-F1	0.4859	—	HGG 0.848/LGG 0.124
Cohen’s κ	−0.0023	—	No better-than-chance agreement
MCC	−0.0026	—	No meaningful correlation
HGG recall	90.85% (745/820)	[88.69, 92.64]	Majority class
LGG recall	8.96% (19/212)	[5.81, 13.57]	Near-absent detection
HGG precision	79.42% (745/938)	[76.72, 81.89]	≈class prevalence
LGG precision	20.21% (19/94)	[13.34, 29.43]	—
McNemar vs. baseline	χ^2^ = 32.18	*p* = 1.4 × 10^−8^	Descriptive only; slice-level observations are not independent

## Data Availability

BraTS2020, BRISC2025 and RadGenome-Brain MRI are publicly available from their respective providers. All code, configuration files, dataset partition files, per-case metric logs and statistical analysis scripts generated by this study will be deposited in a public repository with a permanent identifier, and the identifier will be provided prior to publication.
